# Common Gene Variants in the Tumor Necrosis Factor (TNF) and TNF Receptor Superfamilies and NF-kB Transcription Factors and Non-Hodgkin Lymphoma Risk

**DOI:** 10.1371/journal.pone.0005360

**Published:** 2009-04-24

**Authors:** Sophia S. Wang, Mark P. Purdue, James R. Cerhan, Tongzhang Zheng, Idan Menashe, Bruce K. Armstrong, Qing Lan, Patricia Hartge, Anne Kricker, Yawei Zhang, Lindsay M. Morton, Claire M. Vajdic, Theodore R. Holford, Richard K. Severson, Andrew Grulich, Brian P. Leaderer, Scott Davis, Wendy Cozen, Meredith Yeager, Stephen J. Chanock, Nilanjan Chatterjee, Nathaniel Rothman

**Affiliations:** 1 Division of Cancer Epidemiology and Genetics, National Cancer Institute, National Institutes of Health (NIH), Department of Health and Human Services (DHHS), Rockville, Maryland, United States of America; 2 Mayo Clinic, College of Medicine, Rochester, Minnesota, United States of America; 3 Department of Epidemiology and Public Health, Yale School of Medicine, New Haven, Connecticut, United States of America; 4 School of Public Health, The University of Sydney, Sydney, New South Wales, Australia; 5 University of New South Wales Cancer Research Centre, Prince of Wales Clinical School, University of New South Wales, Sydney, New South Wales, Australia; 6 Department of Family Medicine and Karmanos Cancer Institute, Wayne State University, Detroit, Michigan, United States of America; 7 National Centre in HIV Epidemiology and Clinical Research, University of New South Wales, Sydney, New South Wales, Australia; 8 Fred Hutchinson Cancer Research Center and University of Washington, Seattle, Washington, United States of America; 9 Norris Comprehensive Cancer Center, University of Southern California, Los Angeles, California, United States of America; 10 Core Genotyping Facility, Advanced Technology Center, National Cancer Institute, National Institutes of Health (NIH), Department of Health and Human Services (DHHS), Gaithersburg, Maryland, United States of America; Cleveland Clinic, United States of America

## Abstract

**Background:**

A promoter polymorphism in the pro-inflammatory cytokine tumor necrosis factor (*TNF*) (*TNF* G-308A) is associated with increased non-Hodgkin lymphoma (NHL) risk. The protein product, TNF-α, activates the nuclear factor kappa beta (NF-κB) transcription factor, and is critical for inflammatory and apoptotic responses in cancer progression. We hypothesized that the TNF and NF-κB pathways are important for NHL and that gene variations across the pathways may alter NHL risk.

**Methodology/Principal Findings:**

We genotyped 500 tag single nucleotide polymorphisms (SNPs) from 48 candidate gene regions (defined as 20 kb 5′, 10 kb 3′) in the TNF and TNF receptor superfamilies and the NF-κB and related transcription factors, in 1946 NHL cases and 1808 controls pooled from three independent population-based case-control studies. We obtaineded a gene region-level summary of association by computing the minimum p-value (“minP test”). We used logistic regression to compute odds ratios and 95% confidence intervals for NHL and four major NHL subtypes in relation to SNP genotypes and haplotypes. For NHL, the tail strength statistic supported an overall relationship between the TNF/NF-κB pathway and NHL (p = 0.02). We confirmed the association between *TNF*/*LTA* on chromosome 6p21.3 with NHL and found the *LTA* rs2844484 SNP most significantly and specifically associated with the major subtype, diffuse large B-cell lymphoma (DLBCL) (p-trend = 0.001). We also implicated for the first time, variants in *NFKBIL1* on chromosome 6p21.3, associated with NHL. Other gene regions identified as statistically significantly associated with NHL included *FAS*, *IRF4*, *TNFSF13B*, *TANK*, *TNFSF7* and *TNFRSF13C*. Accordingly, the single most significant SNPs associated with NHL were *FAS* rs4934436 (p-trend = 0.0024), *IRF4* rs12211228 (p-trend = 0.0026), *TNFSF13B* rs2582869 (p-trend = 0.0055), *TANK* rs1921310 (p-trend = 0.0025), *TNFSF7* rs16994592 (p-trend = 0.0024), and *TNFRSF13C* rs6002551 (p-trend = 0.0074). All associations were consistent in each study with no apparent specificity for NHL subtype.

**Conclusions/Significance:**

Our results provide consistent evidence that variation in the TNF superfamily of genes and specifically within chromosome 6p21.3 impacts lymphomagenesis. Further characterization of these susceptibility loci and identification of functional variants are warranted.

## Introduction

There is now convincing evidence that a promoter polymorphism in the pro-inflammatory cytokine tumor necrosis factor (*TNF*) (*TNF* G-308A) is associated with increased risk for non-Hodgkin lymphoma (NHL) and specifically with the NHL subtype, diffuse large B-cell lymphoma (DLBCL) [1], [2]. Further evaluation of genetic variations in *TNF* and lymphotoxin-alpha (*LTA*), the two prototypic genes that form the *TNF* superfamily, suggest a role for additional polymorphisms in NHL risk [3] and support the general importance of this genetic region in NHL and DLBCL risk. There is also preliminary but growing evidence that other genetic variants within the *TNF* and *TNF* receptor (TNFR) superfamilies affect NHL risk [4]–[6].

The TNF gene product, TNF-alpha (TNF-α), is a proinflammatory cytokine involved in a number of biochemical pathways, including the activation of the nuclear factor kappa beta (NF-κB) transcription factor. NF-κB acts in two ways that are relevant for lymphomagenesis. First, it has anti-apoptotic properties and prevents cell death among cells with malignant potential. Second, NF-κB stimulates the immune response, specifically the production of pro-inflammatory cytokines, which permits survival and proliferation of these cells [7]. These roles are supported by mouse models that implicate both NF-κB and TNF-α in tumor promotion [8], [9].

Constitutive NF-κB expression characterizes a number of lymphomas [10]. It is a hallmark of the activated B-cell like (ABC) DLBCL [11], [12], primary mediastinal B-cell lymphomas (PMBLs), primary effusion lymphoma (PEL) [13], [14], and mucosa-associated lymphoid tissue (MALT) lymphoma [15]. NF-κB is also constitutively expressed in Hodgkin lymphoma (HL) cell lines. Human lymphomagenic viruses including the Epstein Barr Virus (EBV), Kaposi sarcoma–associated herpesvirus (KSHV), and human T-lymphotropic virus type I (HTLV-1) also carry NF-κB–activating oncoproteins. As KSHV is a cause of primary effusion lymphoma and HTLV-1 infection causes adult T-cell lymphoma/leukemia, it is not surprising that both lymphomas also have high levels of NF-κB activity [16].

*TNF* and *LTA* form the foundation of the TNF superfamily, a family of homologous genes involved in NF-κB-mediated cellular proliferation, apoptosis, survival and cell differentiation. To our knowledge, there are no published reports specifically targeted at extensive coverage of *TNF* or NF-κB genetic variations and NHL risk.

To address this gap in knowledge, we conducted a pooled analysis comprising three independent population-based case-control studies of NHL to investigate the etiologic relevance of common genetic variations in 48 genes (500 SNPs) encoding important members of the TNF and TNF receptor (TNFR) superfamilies, and NF-kB and related transcription factors, and I-kappa-B proteins and kinases critical for NF-kB activation and mediating its inhibition [7], [17], [18]. Gene families and regions evaluated are denoted in Table 1 and extensively annotated in Supplemental Materials (Tables S1 and S2).

**Table 1:** Gene families and regions of analyzed candidate genes in three independent NHL case-control studies.

Gene family (of 500 SNPs)	Gene regions*
TNF and TNF superfamily (n = 122)	*LTA/TNF, TNFSF4, TNFSF7, TNFSF8, TNFSF9, TNFSF10, TNFSF12, TNFSF13B, TNFSF14, TNFSF18,*
TNF receptors (TNFR) and TNFR superfamily (n = 146)	*TNFRSF1A/LTBR/TNFRSF7, TNFRSF8/TNFRSF1B, TNFRSF9, TRFRSF12A, TNFRSF13B, TNFRSF13C, TNFRSF14, TNFRSF17, CD40, TRADD*
Death receptors (n = 63)	*TNFRSF10B/TNFRSF10C/TNFRSF10D, TNFRSF10A, TNFRSF25*
Fas (n = 44)	*FAS, FASL, FADD, CFLAR*
TRAF family (n = 28)	*TRAF2, TRAF5, TRAF6, TANK*
NFKB complex and transcription factors (n = 51)	*NFKB1, NFKB2, REL, RELA, RELB, IRF4*
I-kappa-B proteins and kinases (n = 47)	*CHUK, IKBKB, NFKBIA, NFKBIE, NFRKB*

Detailed annotation of genes and SNPs shown in Supplemental Materials (Tables S2 and S3).

* Gene regions defined as 20 kb 5′ and 10 kb 3′ of target gene.

## Results

Descriptive characteristics of the 1,946 cases and 1,808 controls by study are provided in Supplemental Materials (Table S3). The distributions of cases and controls were similar with respect to age and race/ethnicity. We note that our pooled population largely comprised non-Hispanic Caucasians (87% of controls, 90% of cases). The National Cancer Institute-Surveillance, Epidemiology, and End Results (NCI-SEER) and Connecticut studies had similar distributions of NHL subtypes, while the New South Wales (NSW) study had a higher frequency of follicular lymphoma (FL) and a lower frequency of chronic lymphocytic leukemia (CLL)/small lymphocytic lymphoma (SLL) and NHL not otherwise specified (NOS), compared to the other two studies.

### Pathway- and gene region-based associations

We first assessed the overall strength of association between all genes in the TNF and NF-kB family of genes with NHL and NHL subtypes by measuring the tail strength statistic, as described in the Methods Section. For NHL and all four subtypes, the tail strength statistics were positive with the most notable tail strength statistic observed for all NHL (p = 0.02), supporting the overall relationship between the TNF/NF-κB pathway and NHL (Table 2).

**Table 2:** Significance levels (p values) for the TNF/NFkB pathway and for each target region calculated from the permutation test [based on 10,000 permutations and the minimum p-trend within each region], for the association with NHL and NHL subtypes (DLBCL, follicular, marginal zone, and CLL/SLL).

	NHL		DLBCL		Follicular		CLL/SLL		Marginal Zone	
	# SNPs p<0.05/Total # SNPs	p	# SNPs p<0.05/Total # SNPs	p	# SNPs p<0.05/Total # SNPs	p	# SNPs p<0.05/Total # SNPs	p	# SNPs p<0.05/Total # SNPs	p
TNF/NFkB pathway^1^	42/500	0.02	35/500	0.065	38/500	0.101	37/500	0.132	30/500	0.081
***By gene region*** 2										
*FAS*	12/23	0.04	2/23	0.1	9/23	0.006	1/23	0.4	4/23	0.3
*IRF4*	3/15	0.03	2/15	0.09	0/15	0.6	2/15	0.2	2/15	0.3
*TNFSF13B*	4/19	0.03	2/19	0.04	2/19	0.2	3/19	0.001	0/19	0.7
*LTA/TNF*	8/17	0.07*	9/17	0.02	3/17	0.07	0/17	0.9	4/17	0.1
*TANK*	1/10	0.02	0/10	0.4	1/10	0.2	2/10	0.2	0/10	0.7
*TNFRSF13C*	1/4	0.03	0/4	0.5	1/4	0.1	0/4	0.3	1/4	0.04
*TNFSF7*	3/14	0.03	3/14	0.04	3/14	0.02	0/14	0.5	0/14	0.7
*NFKBIE*	1/10	0.09**	0/10	0.3	1/10	0.3	2/10	0.2	1/10	0.4

Genes selected are those with permutation p<0.05; results for all genes shown in Supplemental Materials (Table S4).

1 p-value from tail-strength test.

2 p value from minP test.

* likelihood ratio test (LRT) p = 0.0079.

** LRT p = 0.049.

We focus our remaining discussion on eight selected gene regions where the significance levels were <0.05 by minP test or likelihood ratio test, for all NHL. Briefly, as defined in the Methods section, the minP test provides a gene region-level summary of association by assessing statistical significance of the smallest p-trend within each gene region by permutation-based resampling methods and automatically adjusts for the number of tag SNPs tested within that gene region and the underlying linkage disequilibrium pattern. The likelihood ratio test is a complementary test that assesses the relative improvement in model fit from the inclusion of parameters for all independent SNPs in a particular gene region, thus determining if that region might still be implicated in NHL risk despite no apparent significant SNP association. Six of the gene regions (*FAS*, *IRF4*, *TNFSF13B*, *TANK*, *TNFRSF13C*, and *TNFSF7*) were significant at p<0.05 for all NHL by both the minP test and likelihood ratio test; two (*LTA/TNF* and *NFKBIE*) were significant by likelihood ratio test only. We note that six of the eight gene regions selected are within the TNF or TNF receptor (TNFR) superfamilies.

In gene region-based analyses by NHL subtype, we found DLBCL associated with gene variants in the TNF superfamily (*TNFSF13B, LTA/TNF* and *TNFSF7*) (Table 2). Other notable subtype-specific associations include *FAS* and follicular lymphoma (minP = 0.006) and *TNFSF13B* and CLL/SLL (minP = 0.001). Results were generally consistent between the permutation test (p) and likelihood ratio test (global p); results from both tests for all genes are shown in Supplemental Materials (Table S4).

### Association by SNPs

**Figure 1:**
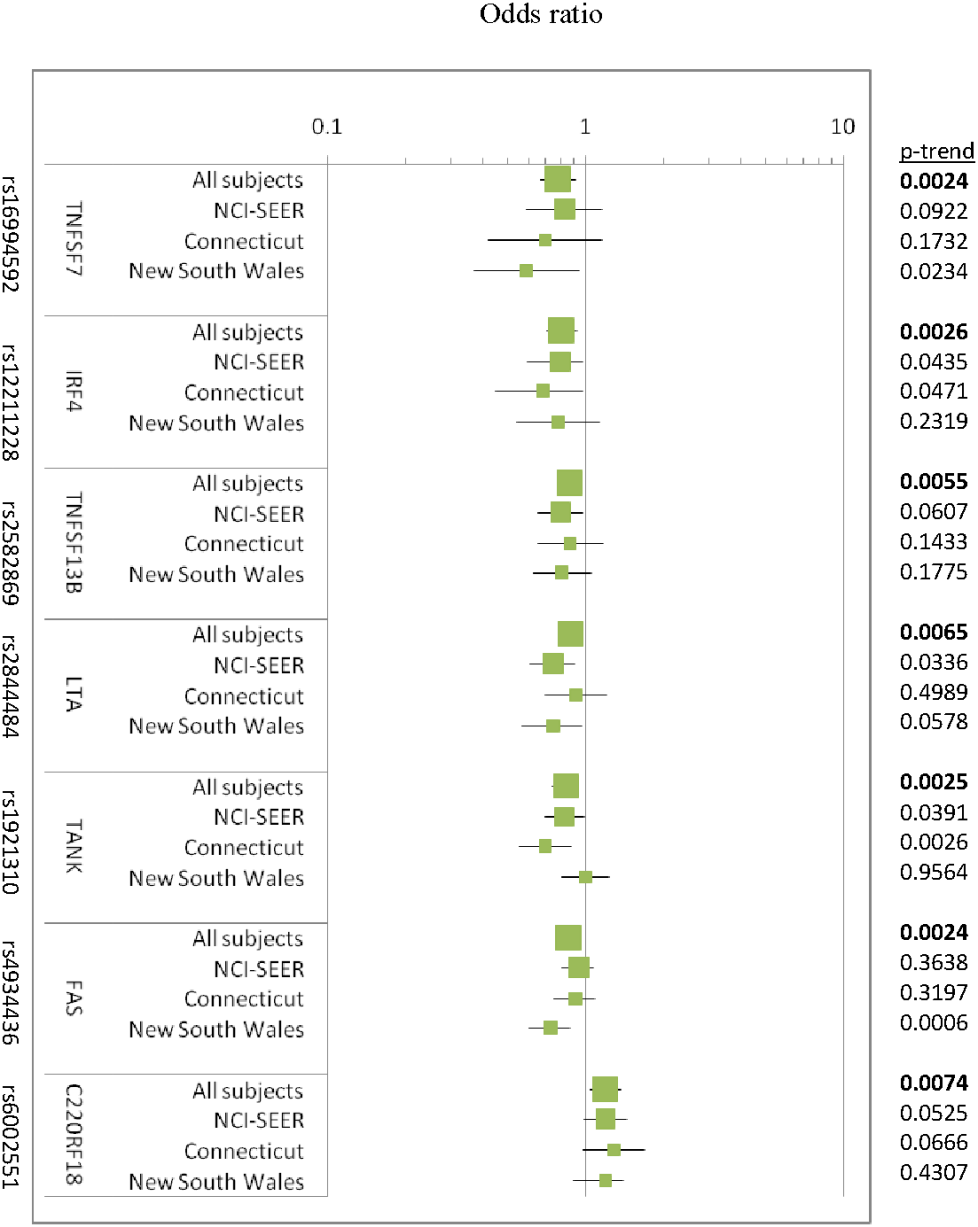
Odds ratios, 95% confidence intervals of additive model for SNPs with p-trends<0.01 for NHL in pooled analyses; data also shown by study (adjusted for age, sex, race, and study site, when applicable). Data for all SNPs (pooled and by study) are shown in Supplemental Materials (Table S5).

**Figure 2:**
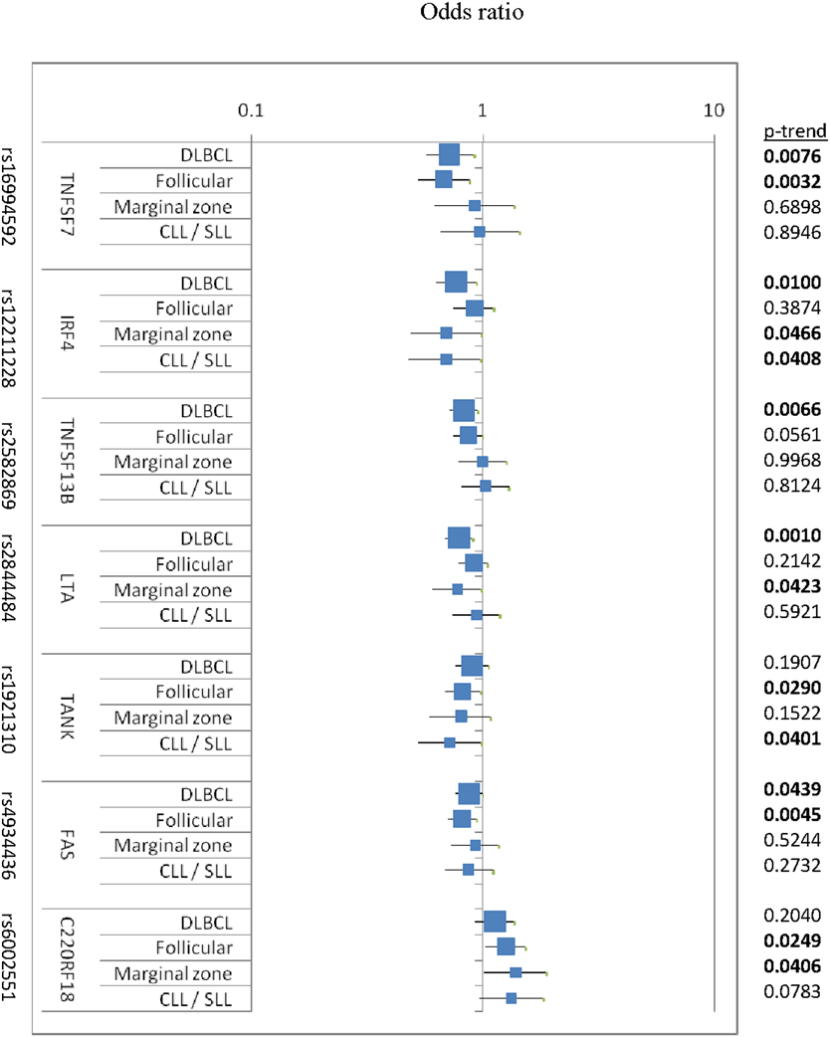
Odds ratios, 95% confidence intervals of additive model for SNPs with p-trends<0.01 for NHL in pooled analyses; data shown by NHL subtypes DLBCL, follicular, CLL/SLL and marginal zone lymphomas in pooled population (adjusted for age, sex, race, and study site). Data for all SNPs by NHL subtype are shown in Supplemental Materials (Table S6).

Consistent with our gene region-based analyses, we found evidence of an altered risk for one or more SNPs in *FAS*, *IRF4*, *TNFSF13B*, *TNFRSF13C*, *TANK*, and *TNFSF7*, for all NHL (Table 2). Among the SNPs with the minimum p value for each gene region, the associations as evaluated using the additive model were consistent across all three independent NHL studies (Figure 1) and were statistically significant for one or more of the most common subtypes (Figure 2). Most associations were found to be statistically significant in at least one of the three studies (e.g., *LTA, FAS, C22ORF13/TNFRSF13C*) and some in two of three studies (e.g., *IRF4, TANK*). The p-trends for each gene variant contributing the minimum p are shown in Figure 1 and ranges from 0.002 to 0.007.

In general, the SNP-based associations were in the same direction for all four subtypes and the calculated p-heterogeneity by the four subtypes was not statistically significant for any SNP (Figure 2). We note that statistically significant associations with DLBCL were observed for SNPs in *TNFSF13B*, *LTA/TNF*, *TNFSF7*, *FAS* and *IRF4*. In addition, *TNFSF7*, *TNFSF13B* and *FAS* were also significantly or marginally significantly associated with follicular lymphoma. Finally, though *TANK* and *C22ORB18/TNFRSF13C* were both statistically significantly associated with follicular and marginal zone only, their associations were in the same direction as for DLBCL and CLL/SLL.

Results for all SNPs and NHL, pooled, by study, and by NHL subtypes are shown in Supplemental Materials (Tables S5 and S6).

### Haplotype associations

Results from haplotype-based analyses (defined by blocks of linkage disequilibrium) were generally consistent with the gene region- and SNP-based findings (data not shown). However, evaluation of the *TNFSF13B* and *TNF/LTA* regions, using a sliding window approach of three loci, suggested new evidence of association for regions not previously implicated with NHL risk in the SNP-based analyses alone. Briefly, evaluation of the *TNFSF13B* region yielded a new region comprising *TNFSF13B* [rs8181791–rs16972216–rs17499386] significantly associated with NHL (p = 0.0025) not originally found significant by SNP-based analysis and not in linkage disequilibrium with the already implicated SNP, *TNFSF13B* rs2582869 (Figure S1).

Of the 16 polymorphisms we evaluated in the *TNF/LTA* region on chromosome 6p21.3 which included the genes *TNF*, *LTA* and *NFKBIL1*, a number of SNPs were associated with NHL, DLBCL, and marginal zone lymphoma. Evaluation of the region using a sliding window of three loci revealed the *NFKBIL1* region to also be significantly associated with NHL, including follicular lymphoma. The previously unidentified SNPs that comprise the *NFKBIL1* haplotype [rs2857605–rs2239707–rs2230365] were most significantly associated with NHL risk (p = 0.0005) (Figure S2).

## Discussion

In our pooled analysis of three predominantly Caucasian studies, we found our set of candidate TNF and NF-kB family of genes to be statistically significantly associated with NHL risk when assessed globally as a pathway. Within the pathway, we found that gene variants largely in the TNF superfamily were associated with NHL, but that there were limited associations with NHL for genetic variation in the NF-κB genes. Specifically, we confirm that genetic variants in *TNF/LTA* (encoding for the proinflammatory cytokines TNF-α and LT-α) were most pronounced for DLBCL and marginal zone lymphoma. We further provide new evidence using haplotype analysis that additional variants in chromosome 6p21.3 (*NFKBIL1-*encoding IKBL1 and thought to be a negative regulator of NF-kappa-B activation) may be important for NHL risk, thus further implicating this chromosomal region in which the search for causal SNPs should be pursued. All other associations reported were in general consistent across all four subtypes evaluated.

A major strength of the present study is the inclusion of data from three independent population-based studies. By pooling these studies, we achieved a sample size providing reasonable power to detect moderate SNP effects, explore subtype specificity and most importantly, evaluate the consistency of results between studies. To our knowledge, the present analysis is the largest evaluation to date specifically evaluating the role of the TNF/NF-κB pathway in NHL risk. We note that our results are consistent when restricted to non-Hispanic Caucasians and thus are unlikely to be biased by population stratification. We acknowledge that some of our findings may be false positive. However, consistency of association across the three studies was a key criterion for identifying our strongest findings, thus reducing this possibility. Nevertheless, our results require replication in other independent populations and further investigation to identify causal variants for NHL risk.

In addition to the confirmation of the *TNF/LTA* locus as important for DBLCL risk, examination of the related *NFKBIL1* gene on chromosome 6p21.3 revealed intriguing results. Though its function remains unknown, it lies near TNF and at the telomeric end of the major histocompatibility complex. Moreover, it is a known susceptibility locus for rheumatoid arthritis [19]. Coupled with the known associations between *TNF/LTA* gene variants with DLBCL risk, *NFKBIL1*'s location on 6p21.3 further implicates this region in NHL risk. Notably, the *NFKBIL1* haplotype is not correlated with the *TNF/LTA* SNPs and supports that a causal SNP for NHL is located in this region. A further detailed examination of this region is thus also warranted based on our haplotype analysis.

All other implicated genes play critical roles in mediating autoimmunity. Protein products of *FAS*, *TNFSF13B* (*BAFF*), *TNFRSF13C* (*BAFF-R*), *TNF* and *LTA* can all activate the NF-kB pathway, while *TANK* (encodes the TANK protein in the cytoplasm and inhibits TRAF function) mediates the pathway indirectly. Briefly, *FAS* (encodes the Fas receptor and binds to the Fas ligand) is a prototypical death receptor and induces caspase-dependent cell death and production of proinflammatory cytokines including TNF-alpha [20], [21]. Fas-deficient mice develop elevated levels of serum autoantibodies and B cell plasmacytoid lymphomas. Patients with autoimmune lymphoproliferative syndrome or ALPS have Fas mutations and are at high risk for NHL [22]. *TNFSF13B* (encodes the cytokine BAFF) and its main receptor BAFF-R (encoded by *TNFRSF13C*) play a central role in peripheral B-cell survival and maturation, including inducing class switch recombination. Altered BAFF expression is associated with autoimmune conditions, B-cell lymphomas and immunodeficiency [23]. *BAFF* and *BAFF-R* knock-out mice have B cells with impaired survival while mice with overexpression of Baff develop mature B-cell hyperplasia, follicular lymphoma, marginal zone lymphoma and symptoms of systemic lupus erythematosus. Immunodeficient mice express a naturally mutated form of BAFF-R [24]. In humans, overexpression of the BAFF protein is found in systemic lupus erythamatosus, rheumatoid arthritis and Sjogren's syndrome, all known risk factors for NHL. Human NHL, CLL and Waldenstrom's macroglobulinaemia B cells also express BAFF-R [25]. In clinical studies, BAFFR-Ig has been shown to inhibit myeloma cell groth in both in vivo (cell culture) and ex vivo (mice) models [26]; further, a fusion toxin has been developed for BLyS receptors such as BAFF-R with cytotoxic effects inhibited when pretreated with soluble BAFF-R decoy receptors [27] A SNP in the promoter of *BAFF* (-871C->T, rs9514827) was previously associated with increased BAFF transcription and the -871T allele was more prevalent in familial CLL patients compared to controls [28]. Though we did not evaluate this SNP, we note that the global test of this gene region was most strongly associated with CLL/SLL. Our results are thus consistent with other reports supporting a role for *BAFF* and its main receptor, *BAFF-R*, as a susceptibility locus for NHL risk.

Our results showing a lack of association for several key NF-κB genes (*CHUK*, which encodes a component of a protein complex that inhibits NF-kB; *IKBKB*, which encodes a protein that is part of the IKK complex and phosphorylates NF-kB inhibitors; and *REL*, a proto-oncogene that encodes the transcription factor c-Rel and binds the NFKB inhibitor) are consistent with a recent study, although we were not able to replicate an association with *NFKB1*, which encodes a pleiotropic transcription factor, reported in that study [6].

Among NF-κB induced genes, we identified *IRF4*, a transcriptional activator that binds to the MHC class I promoter and immunoglobulin lambda light chain enhancer, as relevant for NHL risk. IRF4 is critical for class switch recombination and antibody maturation [29]. Its expression disables germinal center formation by binding to and thus repressing BCL6 [30]–[33]. *IRF4* knock-out mice are unable to mount antibody or antitumor responses. Although high levels of IRF4 expression (and constitutive NF-κB activation) have been reported specifically for the activated B-cell subtype of DLBCL, our results suggest that common variations in *IRF4* may also influence risk for CLL/SLL and MZ, in addition to DLBCL. A recent GWAS also identified *IRF4* (rs872071) as a top hit for CLL, with risk alleles associated with lower mRNA expression in a dose-dependent fashion in the study [34]. Furthermore, in RNA-interference studies, IRF4 inhibition has been demonstrated to be toxic to myeloma cell lines [35]; gene expression profiling also demonstrated IRF4 to target MYC in activated B cells, providing further evidence that aberrant IRF4 regulation is critical for B cell activation.[35] Finally, though downregulation of IRF4 expression has been reported in chronic myeloid leukemia and acute myeloid leukemia patients, IRF4 levels appear to increase during good response to IFN-alpha therapy [36].

In summary, we report consistent evidence in three population-based case-control studies that common genetic variation in genes involved in TNF signaling pathways with NHL risk. Our results are consistent with mouse models that implicate NF-κB and TNF-α in tumor promotion [8], [9]. Further pursuit of functional and causal SNPs within chromosome 6p21.3 and replication of our results are warranted.

## Materials and Methods

### Study Population

Our study population was derived from pooling three independent population-based case-control studies, which have been described in detail previously: the National Cancer Institute-Surveillance Epidemiology and End Results (NCI-SEER) NHL Case-Control Study [37], the Connecticut NHL Case-Control Study [38], [39], and the New South Wales (NSW) NHL Case-Control Study [40]. All three studies included first primary NHL cases only, and population controls were frequency matched to cases. The studies were approved by the Institutional Review Boards of the NCI and each SEER center for the NCI-SEER study; Yale University, the Connecticut Department of Public Health, and the NCI for the Connecticut study; and all participating institutions for the NSW study. All study participants provided informed consent.

### NHL Pathology Classification

In the NCI-SEER and NSW study, all cases were histologically confirmed by the local diagnosing pathologist. In the NSW study, an expert hematopathologist also reviewed the pathological material centrally when she judged the diagnosis of NHL to be <90% certain upon review of the original pathology reports (including flow cytometry). In the Connecticut study, all cases were confirmed by central review of diagnostic slides by two independent expert hematopathologists.

For the pooled analyses, we evaluated NHL overall and specific NHL subtypes, grouping cases according to the World Health Organization classification [41] using the International Lymphoma Epidemiology Consortium (InterLymph) guidelines [42]. For analyses by NHL subtype, we evaluated the four most common subtypes: diffuse large B-cell lymphoma (DLBCL) (28%), follicular lymphoma (28%), marginal zone lymphoma (8%), and chronic lymphocytic leukemia/small lymphocytic lymphoma (CLL/SLL) (8%) (Table 2). We note that our studies primarily included SLL rather than CLL cases because these diseases were not considered the same entity until the WHO classification was introduced in 2001.

### Laboratory Methods

#### Biological samples and DNA extraction

Study participants who did not provide a biologic specimen, did not have sufficient material for DNA extraction or sufficient DNA for genotyping, or whose genotyped sex was discordant from the questionnaire data were excluded from this analysis. Specifically, for the NCI-SEER study, of 1231 cases (820 blood, 411 buccal cell) and 992 controls (692 blood, 300 buccal cell) with biospecimens, 1001 cases and 834 controls were genotyped. For the Connecticut study, 436 of 486 cases and 517 of 578 controls with bloods were genotyped. For the NSW study, 524 of 597 cases and 474 of 525 controls with bloods were genotyped.

For the NCI-SEER study, DNA was extracted from blood clots or buffy coats (BBI Biotech, Gaithersburg, MD) using Puregene Autopure DNA extraction kits (Gentra Systems, Minneapolis, MN), and from buccal cell samples by phenol-chloroform extraction methods [43]. Genotype frequencies for individuals who provided blood compared with buccal cells were equivalent [44]. For the Connecticut study, DNA was extracted from the blood samples using phenol-chloroform extraction methods. For the NSW study, DNA was extracted from buffy coats using Qiagen QIAamp® DNA Blood Midi Kits by laboratory staff at the Viral Epidemiology Section, SAIC-Frederick, NCI-Frederick.

#### Genotyping

Genotyping of tag SNPs from 48 candidate gene regions involved in TNF/NF-κB pathway was conducted at the NCI Core Genotyping Facility (Advanced Technology Center, Gaithersburg, MD; http://snp500cancer.nci.nih.gov) [45] using a custom-designed GoldenGate assay (Illumina, www.illumina.com). The GoldenGate assay included a total of 1536 tag SNPs, thus this analysis was conducted as part of a panel that also included SNPs from candidate genes in other pathways. Tag SNPs were chosen from the designable set of common SNPs (minor allele frequency (MAF)>5%) genotyped in the Caucasian (CEU) population sample of the HapMap Project (Data Release 20/Phase II, NCBI Build 35 assembly, dbSNPb125) using the software Tagzilla (http://tagzilla.nci.nih.gov/), which implements a tagging algorithm based on the pairwise binning method of Carlson *et al.* [46]. For each original target gene, SNPs within the region spanning 20 kb 5′ of the start of transcription (exon 1) to 10 kb 3′ of the end of the last exon were grouped using a binning threshold of r^2^>0.8 to define a gene region. When there were multiple transcripts available for genes, only the primary transcript was assessed.

#### Quality control (QC)

We excluded tag SNPs that failed to properly cluster or did not amplify. SNPs with low completion rate (<90% of samples) were excluded by study (NCI-SEER blood samples: N = 1; NCI-SEER buccal cell samples: N = 4). QC duplicates and replicates from each study were genotyped, blinded to laboratory personnel. SNPs with concordance <95% in the study-specific QC samples were excluded for that study (NCI-SEER buccal cell samples: N = 1). We also excluded samples with a low completion rate (<90% of the full panel of 1536 tag SNPs; NCI-SEER: 11 cases, 6 controls; Connecticut: 2 controls; NSW: 4 cases, 9 controls). We included in our pooled analyses, 9 candidate SNPs previously genotyped and analyzed by Taqman assay in at least two of the three studies and located within one of our 48 candidate genes.

Hardy–Weinberg equilibrium was evaluated among non-Hispanic Caucasian controls (N = 1578, 87% of the analytic population) for the pooled study population and by study. SNPs showing evidence of deviation from Hardy–Weinberg proportions (p<0.0001) included *FAS* rs1051070, *TNFSF13B* rs1041569, *TNFSF10* rs2041693, *TNFRSF8* rs11569835, *NFKBIA* rs17103286, *TNFRSF1B* rs1061624, *CD40* rs11569309, *TNFRSF13B* rs7504096, and *TNFRSF10C* rs12545733. Though our QC data did not suggest any obvious genotyping error and we present their results, we note caution in interpretation of these select results.

In total, we evaluated 48 *a priori* candidate gene regions selected from the TNF/NF-κB pathway (Table 1, Table S1). Specifically, the TNF/NF-κB pathway comprised genes from TNF and the TNF superfamily, TNF receptors (TNFR) and the TNFR superfamily, the TNF receptor-associated factor family (TRAF), the NFKB complex, transcription factors, and the NFKB related I-Kappa-beta proteins (Table 1).

#### Final analytic population

The final pooled analytic study population included 1,946 cases and 1,808 controls (NCI-SEER: 990 cases, 828 controls; Connecticut: 436 cases, 515 controls; NSW: 520 cases, 465 controls) with data for 500 SNPs (491 tag SNPs and 9 previously genotyped Taqman SNPs).

### Statistical methods

#### Pathway-based analysis

We summarized the overall evidence of association for the 500 SNPs with NHL or an NHL subtype by using the “tail strength” statistic [47], a summary measure for the departure of the observed p-value distribution from their expected distribution under the global null hypothesis of no association in the whole pathway (all 48 candidate gene regions in this analysis). We assessed the significance of the tail strength statistics by generating their null distributions by permutation-based resampling of the data. Analyses were conducted using the MATLAB Statistics Toolbox™ 6.2 (The Mathworks, Inc., Natick, MA).

#### Gene region-based analyses

We obtained a gene region-level summary of association using two methods. First, we computed the minimum p-value (“minP test”), which assesses the statistical significance of the smallest p-trend within each gene region (determined by dichotomous logistic regression, comparing NHL or NHL subtypes to controls) by permutation-based resampling methods (10,000 permutations) that automatically adjust for the number of tag SNPs tested within that gene and the underlying linkage disequilibrium pattern [48], [49]. To account for multiple comparisons within the pathway (all 48 candidate gene regions in this analysis), we applied the false discovery rate (FDR) method of Benjamini and Hochberg [50] to the minP test separately for NHL and each subtype.

We also conducted a likelihood ratio test, assessing the relative improvement in model fit from the inclusion of parameters for all independent SNPs (r^2^<0.8 among controls) in a particular gene, assuming a codominant model for each SNP. Unlike the minP test which ascertains significance of a genetic region with NHL risk based on the minimum p value after adjusting for the total number of SNPs evaluated in the region, this second method was used to determine if additional genetic regions might be implicated in NHL risk despite no apparent significant SNP association.

#### SNP-based analyses

We calculated odds ratios (OR) and 95% confidence intervals (CI) estimating the relative risk of NHL and NHL subtypes in relation to SNP genotype using dichotomous and polytomous unconditional logistic regression models, respectively. The homozygote of the most common allele in the pooled study population was used as the referent group. Tests for trend under the co-dominant model used a three-level ordinal variable for each SNP (0 = homozygote common, 1 = heterozygote, 2 = homozygote variant). All models were adjusted for age, race/ethnicity, sex, and study center (categories listed in Table 2). We conducted analyses restricted to non-Hispanic Caucasians and stratified by age (<50, ≥50 years) and sex to evaluate the consistency of our results by various demographic groups. To evaluate the consistency of our results by NHL subtype, we assessed heterogeneity among NHL subtypes in the polytomous multivariate unconditional logistic regression models using the Wald chi-square statistic. Analyses were conducted using SAS version 9.1 (SAS Institute, Cary, NC).

#### Haplotype analyses

We conducted haplotype analyses among non-Hispanic Caucasians using two methods. First, we evaluated risk of NHL and NHL subtypes associated with haplotypes defined by SNPs within a sliding window of three loci across a gene (Haplo Stats, version 1.2.1, haplo.score.slide, http://mayoresearch.mayo.edu/mayo/research/schaid_lab/software.cfm). A global score statistic was used to summarize the evidence of association of disease with the haplotypes for each window. Second, we visualized haplotype structures using Haploview, version 3.11 [51] based on measures of pairwise linkage disequilibrium between SNPs. For blocks of linkage disequilibrium, we obtained ORs and 95% CIs for the underlying haplotypes under the assumption of an additive model (haplo.glm, minimum haplotype frequency 1%). All haplotype analyses were adjusted for age, sex, and study center.

## Supporting Information

Table S1 Supplemental Table 1
(0.09 MB DOC)

Table S2 Supplemental Table 2
(0.67 MB DOC)

Table S3 Supplemental Table 3
(0.10 MB DOC)

Table S4 Supplemental Table 4
(0.11 MB DOC)

Table S5 Supplemental Table 5
(2.34 MB XLS)

Table S6 Supplemental Table 6
(2.33 MB XLS)

Figure S1 Supplemental Figure 1
(0.06 MB PDF)

Figure S2 Supplemental Figure 2
(0.06 MB PDF)
