# Supplementary material for: Common Gene Variants in the Tumor Necrosis Factor (TNF) and TNF Receptor Superfamilies and NF-kB Transcription Factors and Non-Hodgkin Lymphoma Risk
Source: PLoS One. 2009 Apr 24;4(4):e5360. doi: 10.1371/journal.pone.0005360 (PMC2669130; doi:10.1371/journal.pone.0005360)
Supplement: Table S1 — Supplemental Table 1 (0.09 MB DOC) [file pone.0005360.s001.doc]

Supplemental Table 1. Tumor necrosis factor (TNF) and nuclear factor kappa beta (NFKB) regions targeted, reflecting 48 *a priori* candidate genes evaluated in risk of NHL and four NHL subtypes across three independent NHL case-control studies*. Genotyping data validated for 500 SNPs (491 Illumina GoldenGate and 9 TaqMan**).

| **Target**  **Regions** | **A Priori Candidate Genes**  **Gene Name (Alias)** | **All genes included in region**  **(# SNPS validated/total # SNPs***), [TaqMan SNPs]** | **Relationship of candidate gene to TNF and/or NFkB pathway****** |
| --- | --- | --- | --- |
| **TNF and TNF SUPERFAMILY** | | | |
| *LTA/*  *TNF* | Lymphotoxin-alpha (TNFB)  Tumor necrosis factor-alpha (TNFA) | NFKBIL (6/6)  LTA (3/3), [2]  TNF (1/1), [4]  LST1 (1/1) | LTA shares receptor with TNF and binds to both TNFR1 and TNFR2. LTA accelerates initiation of immune responses and protects the host from larger viral challenges  Proinflammatory cytokine |
| *TNFSF12* | Tumor necrosis factor ligand superfamily, member 12 (TWEAK) | TNFSF12 (1/1)  SENP3 (2/2) | Binds to DR3 and induces apoptosis and NF- kappa-B activation. Overlapping signaling functions with TNF, but displays a much wider tissue distribution. |
| *TNFSF10* | Tumor necrosis factor ligand superfamily, member 10 (TRAIL, APO2L) | TNFSF10 (17/17) | Induces apoptosis but mediated through TRAIL receptors TRAILR1 (DR4) and TRAILR2 (DR5). |
| *TNFSF14* | Tumor necrosis factor ligand superfamily, member 14 (HVEML) | TNFSF14 (3/3)  C3 (11/11) | Activates NFKB and stimulates proliferation of T cells. |
| *TNFSF13B* | Tumor necrosis factor ligand superfamily, member 13B (BLYS, BAFF, TALL1, THANK) | TNFSF13B (17/17)  C13orf6 (2/2) | Cytokine that activates both nuclear factor kappa-B and induces apoptosis in activated T cells.  Enhances B-cell survival in vitro and is a regulator of the peripheral B-cell population. Overexpression in mice results in mature B-cell hyperplasia and symptoms of systemic lupus erythematosus Like APRIL (TNFSF13), BAFF binds to both TACI and BCMA (TNFRSF17). BAFFR is the principal receptor required for BAFF-mediated mature B-cell survival |
| *TNFSF18* | Tumor necrosis factor ligand superfamily, member 18 (AITRL, GITRL) | TNFSF18 (4/4) | Members of the TNF superfamily, TNFSF18, regulates diverse biologic functions, including cell proliferation, differentiation, and survival. Activates the antiapoptotic transcription factor NF-kappa-B |
| *TNFSF4* | Tumor necrosis factor ligand superfamily, member 4 (OX40L, GP34, CD134L) | TNFSF4 (11/11) | Required for dendritic cell-mediated antitumor immunity. |
| *TNFSF7* | Tumor necrosis factor ligand superfamily, member 7 (CD70, CD27L) | TNFSF7 (14/14) | CD27L is homologous to the ligands of the TNF receptor family, including TNF-alpha, TNF-beta, and the CD40 ligand, CD27L. Induces proliferation of costimulated T cells and enhanced generation of cytolytic T cells. |
| *TNFSF8* | Tumor necrosis factor ligand superfamily, member 8 (CD30LG, CD153) | TNFSF8 (15/16) | CD30L induces cell death; receptor-binding region shares similarity with TNF-alpha, TNF-beta and CD40LG. |
| *TNFSF9* | Tumor necrosis factor ligand superfamily, member 9 | TUBB4 (3/3)  TNFSF9 (5/5) | Enhances primary T-cell proliferation and activation-induced cell death in long-term T-cell clones. |

*Supplemental Table 1 (continued)*

| **Gene/Regions targeted** | **Gene name (Alias)** | **Genes included**  **(# SNPS validated/total # SNPs**), [TaqMan SNPs]** | **Relation to TNF and/or NFkB pathway** |
| --- | --- | --- | --- |
| **TNF RECEPTORS (TNFR) AND TNFR SUPERFAMILY** | | | |
| *TNFRSF1A/*  *LTBR/*  *TNFRSF7* | Tumor necrosis factor receptor superfamily, member 1A (TNFR1, TNFAR)  Lymphotoxin B receptor (TNFCR)  Tumor necrosis factor superfamily, member 7 (CD27, S152) | TNFRSF1A (5/5)  SCNN1A (7/7)  LTBR (3/3)  LOC39028 (5/5)  TNFRSF7 (5/6)  TAPBPL (5/5) | LTBR signaling mediated by RELB (NFKB2)-NFKB pathway. Receptor for LTA and LTB, takes part in immunologic reactions, but does not bind to either TNFR1 or TNFR2.  TNFR1-induced apoptosis involves 2 sequential signaling complexes. Complex I, the initial plasma membrane-bound complex, consists of TNFR1, TRADD, RIP1, and TRAF2 and rapidly signals activation of NF-kappa-B. In a second step, TRADD and RIP1 associate with FADD and caspase-8, forming a cytoplasmic complex, complex II. When NF-kappa-B is activated by complex I, complex II harbors the caspase-8 inhibitor FLIP-L and the cell survives. Thus, TNFR1-mediated signal transduction includes a checkpoint, resulting in cell death (via complex II) in instances where the initial signal (via complex I and NF-kappa-B) fails to be activated. |
| *TNFRSF8/*  *TNFRSF1B* | Tumor necrosis factor receptor superfamily, member 8 (CD30)  Tumor necrosis factor receptor superfamily, member 1B (TNFR2, TNFBR) | RP5-1077B9.4 (1/1)  TNFRSF8 (26/27)  TNFRSF1B (14/15)  LOC390998 (3/3) | A member of the TNF receptor superfamily, CD30 is a surface antigen used as a clinical marker for Hodgkin lymphoma and related hematologic malignancies. CD30 signalizing limits proliferative potential of autoreactive CD8 effectorT cells, and protects the body against autoimmunity.  TNFR2 is the main TNF receptor found on circulating T cells and is the major mediator of autoregulatory apoptosis in CD8+ cells. TNFR2 may act with TNFR1 to kill nonlymphoid cells. |
| *TRADD* | Tumor necrosis factor receptor 1-associated death domain protein | LIN10 (2/2)  TRADD (1/1)  NOL3 (2/2) | Forms membrane-bound complex with TNFR1, TRADD, RIP1 and TRAF2 to rapidly signal activation of NF-kappa-B. This activation results in cell survival because complex II harbors the caspase-8 inhibitor FLIP-L.  TRADD and RIP1 also associate with FADD and caspase-8 to form cytoplasmic complex II. This results in cell death when the initial signal via complex I and NF-kappa-B fails to be activated. |
| *CD40* | CD40 antigen (TNFRSF5) | RPL13P2 (2/2)  CD40 (13/13) | Inhibits NFKB activation in conjunction with TRAF2 and TRAF6. |
| *TNFRSF12A* | Tumor necrosis factor receptor superfamily, member 12A (TWEAKR, FN14) | CLDN9 (4/4)  TNFRSF12A (1/1)  WDR58 (1/1) | Receptor for TWEAK; the TWEAK-TWEAKR system plays a role in endothelial cell growth and migration. |
| *TNFRSF13B* | Tumor necrosis factor receptor superfamily, member 13B (TACI) | TNFRSF13B (21/21)  LOC96597 (5/5) | A member of the tumor necrosis factor receptor (TNFR) superfamily, plays role in activation of the transcription factors NFAT, AP1 and NF-kappa-B. |
| *TNFRSF13C* | Tumor necrosis factor receptor superfamily, member 13C (BAFFR) | TNFRSF13C (1/1)  C22orf18 (3/3) | BAFFR binds BAFF, inhibiting BAFF-mediated costimulation of B-cell proliferation. BAFFR thought to be principal receptor required for BAFF-mediated mature B-cell survival |

*Supplemental Table 1 (continued)*

| **Target**  **Regions** | **A Priori Candidate Genes**  **Gene Name (Alias)** | **All genes included in region**  **(# SNPS validated/total # SNPs***), [TaqMan SNPs]** | **Relationship of candidate gene to TNF and/or NFkB pathway****** | |
| --- | --- | --- | --- | --- |
| **TNF RECEPTORS (TNFR) AND TNFR SUPERFAMILY *(continued)*** | | | | |
| *TNFRSF14* | Tumor necrosis factor receptor superfamily, member 14 (HVEM, HVEA, TR2) | TNFRSF14 (3/3) | | A member of the TNFR family. Play key role in regulating the immune response to infection.  Interacts with several TRAF proteins, including TRAF1, 2, 3, 5.  Expression activated NF-kappa-B  Linked via TRAFs to signal transduction pathways that activate the immune response. |
| *TNFRSF17* | Tumor necrosis factor receptor superfamily, member 17 (BCMA, BCM) | TNFRSF17 (6/7)  RUNDC2A (2/2) | | Receptor to BAFF ligand. Consistent with other TNFRs, overexpression induces NFKB activation. |
| *TNFRSF9* | Tumor necrosis factor receptor superfamily, member 9 (CD137, ILA) | TNFRSF9 (5/5) | | A member of the TNF receptor family, important regulators of immune responses. Expressed by activated T and B lymphocytes and monocytes, it inhibits proliferation of activated T lymphocytes and induces programmed cell death. |
| ***Death receptors*** | |  | |  |
| *TNFRSF25* | Tumor necrosis factor receptor superfamily, member 25 (DR3, APO3, LARD, formerly TNFRSF12) | ESPN (1/1)  TNFRSF25 (2/2)  PLEKH5 (4/4) | | Loss thought to be key event in the development of lymphoid malignancies, allowing cells to escape from homeostatic processes that limit lymphocyte proliferation. |
| *TNFRSF10B/*  *TNFRSF10C/*  *TNFRSF10D/*  *TNFRSF10A* | Tumor necrosis factor receptor superfamily, member 10B (DR5, TRAILR2, TRICK2)  Tumor necrosis factor receptor superfamily, member 10C (DCR1, TRAILR3, TRID)  Tumor necrosis factor receptor superfamily, member 10D (DCR2, TRAILR4, TRUNDD)  Tumor necrosis factor receptor superfamily, member 10A (DR4, TRAILR1, APO2) | RHOBTB2 (3/3)  TNFRSF10B (12/12*****)  TNFRSF10C (12/12)  TNFRSF10D (10/10)  TNFRSF10A (13/13), [1]  LOC389641 (3/3)  CHMP7 (2/2) | | Certain cytokines of the tumor necrosis factor (TNF) ligand family induce apoptosis by binding to their respective death domain-containing receptors. Death receptor-4' (DR4) overexpression induces apoptosis independent of FADD. TRAIL receptor DR5 engages in caspase-dependent aoptotic pathway; also mediates apoptosis via FADD. Two decoy receptors for TRAIL, DCR1 (TRAILR3) and DCR2 (TRAILR4) and do not signal apoptosis. |
| ***FAS*** | | | | |
| *FAS* | Tumor necrosis factor receptor superfamily, member 6 (CD95, APO1, APT1) | FAS (23/23), [2] | | Interaction with FAS ligand induces cell death.  Inability of homozygous mutant mice to mediate FAS-induced apoptosis provokes a complex immunologic disorder featuring defects in both the B and T lymphoid compartments.  In humans, FAS mutations result in autoimmune lymphoproliferative syndrome, or ALPS |
| *FASL* | Tumor necrosis factor ligand superfamily, member 6 (CD95L, TNFSF6, CD178, APT1LG1) | FASLG (11/12) | | Part of the TNF family, decreased FASL activity decreases activation-induced cell death, and increased T-cell proliferation after activation. |
| *FADD* | FAS-associated via death domain (MORT1) | FADD (2/2)  TMEM16A (3/3) | | Interacts with FAS; mediates signaling of all known death domain-containing members of the TNF receptor superfamily.  Overexpression induces apoptosis. |
| *CFLAR* | CASP8- and FADD-like apoptosis regulator  (FLIP, I-FLICE, CASPER, CASH, FLAME1, CLARP, MRID) | NDUFB3 (1/1)  CFLAR (2/2) | | Deletion of C-terminal residues inhibits TNF- and FAS-induced apoptosis.  Cooperates with CASP8 and FADD to regulate death factor-induced apoptosis induced by FAS or TNFR1.  Deficiency correlated with activated NFKB. |

*Supplemental Table 1 (continued)*

| **Target**  **Regions** | **A Priori Candidate Genes**  **Gene Name (Alias)** | **All genes included in region**  **(# SNPS validated/total # SNPs***), [TaqMan SNPs]** | **Relationship of candidate gene to TNF and/or NFkB pathway****** | |
| --- | --- | --- | --- | --- |
| **TRAF FAMILY** | | | | |
| *TRAF2* | TNF receptor-associated factor 2 (TRAP) | TRAF2 (4/4) | | TRAF2 is required for NF-kappa-B activation by 2 TNF receptors, TNFR1 and TNFR2. Also interacts with CD30 to mediate activation of nuclear factor kappa-B. |
| *TRAF5* | TNF receptor-associated factor 5 | RCOR3 (1/1)  TRAF5 (4/4)  C1orf97 (1/1) | | TRAF5 hypothesized to active NF-kappa-B activation via lymphotoxin B receptor. Also interacts with CD40 to mediate the activation of nuclear factor kappa-B. |
| *TRAF6* | TNF receptor-associated factor 6 | TRAF6 (3/3)  RAG1 (3/3)  RAG2 (1/1)  LOC119710 (1/1) | | Overexpression of TRAF6 activates NF-kappa-B and activates I-kappa-B kinase in response to proinflammatory cytokines |
| *TANK* | TRAF family member-associated NF-kappa-B activator (ITRAF) | TANK (10/10) | | Blocks TRAF2 binding to inhibit NF-kappa-B activation. |
| **NFKB COMPLEX AND TRANSCRIPTION FACTORS** | | | | |
| *NFKB2* | Nuclear factor kappa-B, subunit 2 (LYT10) | GBF1 (2/2)  NFKB2 (2/2)  PSD (1/1) | | Along with NFKB1, binds to REL, RELA or RELB to form NFKB complex. |
| *NFKB1* | Nuclear factor kappa-B, subunit 1 | NFKB1 (12/12)  MANBA (1/1) | | Along with NFKB2, binds to REL, RELA or RELB to form the NFKB complex. |
| *REL* | V-rel avian reticuloendotheliosis viral oncogene homolog | REL (7/7)  FLJ32312 (1/1) | | Bound by NFKB1 or NFKB2 to form part of the NFKB complex. |
| *RELA* | V-rel avian reticuloendotheliosis viral oncogene homolog A (NFKB3) | SIPA1 (2/2)  RELA (2/2) | | Bound by NFKB1 or NFKB2 to form part of the NFKB complex. The p50 (NFKB1)/p65 (RELA) heterodimer is the most abundant form of NFKB. |
| *RELB* | V-rel avian reticuloendotheliosis viral oncogene homolog B (IREL) | CLPTM1 (3/3)  RELB (2/2)  SFRS16 (1/1) | | Bound by NFKB1 or NFKB2 to form part of the NFKB complex. |
| *IRF4* | Interferon regulatory factor 4 (MUM1, LSIRF) | IRF4 (15/25) | | Important role in B-cell proliferation and differentiation and has oncogenic activity in vitro.  Overexpression thought to contribute to tumorigenesis.  NFKB expression induces IRF4 transcription |

*Supplemental Table 1 (continued)*

| **Target**  **Regions** | **A Priori Candidate Genes**  **Gene Name (Alias)** | **All genes included in region**  **(# SNPS validated/total # SNPs***), [TaqMan SNPs]** | **Relationship of candidate gene to TNF and/or NFkB pathway****** |
| --- | --- | --- | --- |
| **I-KAPPA-B PROTEINS AND KINASES** | |  |  |
| *CHUK* | Conserved helix-loop-helix ubiquitous kinase  (IKBKA, IKKA, IKK1, NFKBIKA) | CHUK (3/3)  CWF19L1 (1/1) | Phosphorylation enables activation of NFKB.  Phosphorylates NFKBIA and NFKBIB, leads to release of NFKB and activation of nuclear genes. Mutant CHUK acts as dominant-negative inhibitor of TNF, IL1, TRAF2, and NIK-induced NFKB activation. |
| *IKBKB* | Inhibitor of kappa light chain gene enhancer in B cells, kinase of, beta (IKKB, IKK2, NFKBIKB) | IKBKB (6/6)  DKK4 (1/1) | A target for profinflammatory stimuli; phosphorylation results in activation of NFKB complex. |
| *NFKBIA* | Nuclear factor of kappa light chain gene enhancer in B cells inhibitor, alpha (NFKBI, IKBA) | NFKBIA (9/9)  DNAJC8P1 (4/4) | One of the I-kappa-B proteins (NFKBIA or NFKBIB), inactivates NF-kappa-B by trapping it in the cytoplasm. IKBA thus mediates NFKB activation and strong negative feedback regulation. |
| *NFKBIE* | Nuclear factor of kappa light chain gene enhancer in B cells inhibitor, epsilon (IKBE) | HSPCB (1/1)  SLC35B2 (2/2)  NFKBIE (3/3)  LOC441151 (1/1)  RP11-444E17.2 (3/3) | Interacts most strongly with RELA and REL but also with NFKB1 and NFKB2. In contrast to NFKBIA, IKBE responds more slowly to IKK activation and acts to dampen long-term oscillations of the NFKB response. |
| *NFRKB* | Nuclear factor related to kappa-B binding protein | TMTM45B (3/3)  NFRKB (6/6)  PRDM10 (4/4) | NFRKB binds to a related variant of the kappa-B site that regulates interleukin-2 receptor alpha-chain gene expression, a critical event in T-cell activation. |

*NCI-SEER NHL case-control study, Connecticut SEER NHL case-control study, New South Wales/Australia case-control study

**17 SNPs failed validation on the Illumina GoldenGate platform

***Number of markers based on r2=0.8 using Tagzilla

****Source: Online Mendelian Inheritance in Man (OMIM) (<http://www.ncbi.nlm.nih.gov/sites/entrez?db=omim>)

*****One SNP excluded due to HWE<0.01
