# Supplementary material for: Common Gene Variants in the Tumor Necrosis Factor (TNF) and TNF Receptor Superfamilies and NF-kB Transcription Factors and Non-Hodgkin Lymphoma Risk
Source: PLoS One. 2009 Apr 24;4(4):e5360. doi: 10.1371/journal.pone.0005360 (PMC2669130; doi:10.1371/journal.pone.0005360)
Supplement: Table S2 — Supplemental Table 2 (0.67 MB DOC) [file pone.0005360.s002.doc]

Supplemental Table 2. TagSNPs genotyped within selected candidate regions (ordered by chromosome) of the TNF and/or NFKB pathway included on the Illumina GoldenGate and analyzed for associations with NHL and four NHL subtypes in three independent case-control studies; additional TaqMan SNPs are noted.

| **Region** | **DB SNP ID** | **Chromosome** | **Chromosomal position** | **Genomic position** |
| --- | --- | --- | --- | --- |
| *FASL* | rs2639621 | 1 | 169341417 | g.-18581T>G |
|  | rs2859228 | 1 | 169344898 | g.-15100A>G |
|  | rs2859234 | 1 | 169350788 | g.-9210C>T |
|  | rs2639614 | 1 | 169357614 | g.Ex2C>T |
|  | rs929087 | 1 | 169363714 | g.IVS2-1417A>G |
|  | rs2639654 | 1 | 169364260 | g.IVS2-871A>C |
|  | rs5030772 | 1 | 169365007 | g.IVS2-124A>G |
|  | rs859663 | 1 | 169369112 | g.Ex4+1443T>G |
|  | rs1492907 | 1 | 169373775 | g.*6961C>T |
|  | rs2021840 | 1 | 169374877 | g.*8063A>G |
|  | rs12085107 | 1 | 169375879 | g.*9065A>T |
| *TNFRSF14* | rs2234167 | 1 | 2522390 | g.Ex7-6G>A |
|  | rs2234161 | 1 | 2525515 | g.IVS3-57C>T |
|  | rs4310388 | 1 | 2539577 |  |
| *TNFRSF25* | rs3138158 | 1 | 6454578 | g.Ex13+254C>T |
|  | rs3138156 | 1 | 6457696 | g.IVS5-223A>G |
|  | rs2986754 | 1 | 6460731 | g.-5756G>C |
|  | rs3007420 | 1 | 6464089 | g.IVS18-87C>G |
|  | rs3007421 | 1 | 6464455 | g.IVS18+107C>T |
|  | rs2986751 | 1 | 6469047 | g.IVS7-134C>T |
|  | rs2986749 | 1 | 6478273 | g.IVS3-6289T>C |
| *TNFRSF8/TNFRSF1B* | rs12042105 | 1 | 12039314 | g.*13182T>C |
|  | rs4644506 | 1 | 12044834 | g.-13087C>G |
|  | rs6541012 | 1 | 12046986 | g.-10935C>T |
|  | rs4427416 | 1 | 12049957 | g.-7964T>C |
|  | rs1318008 | 1 | 12060631 | g.IVS1+2647T>A |
|  | rs12029016 | 1 | 12062764 | g.IVS1+4780G>A |
|  | rs4491070 | 1 | 12064317 | g.IVS1+6333C>T |
|  | rs6690493 | 1 | 12069792 | g.IVS1-8995A>G |
|  | rs2297875 | 1 | 12075727 | g.IVS1-3060A>G |
|  | rs12133231 | 1 | 12082019 | g.IVS2+3145G>A |
|  | rs1201110 | 1 | 12087454 | g.IVS2-3970G>A |
|  | rs11569835 | 1 | 12091554 | g.IVS3+14T>C |
|  | rs11569839 | 1 | 12092147 | g.IVS3+607G>A |
|  | rs10864549 | 1 | 12099303 | g.IVS4+449T>C |
|  | rs12736809 | 1 | 12102724 | g.IVS4-1165T>A |
|  | rs11569864 | 1 | 12102789 | g.IVS4-1100C>T |
|  | rs11569869 | 1 | 12103561 | g.IVS4-328G>A |
|  | rs646249 | 1 | 12114390 | g.IVS8-3217G>A |
|  | rs2486316 | 1 | 12117511 | g.IVS8-96G>T |
|  | rs8179353 | 1 | 12118641 | g.IVS10+491T>C |
|  | rs1201124 | 1 | 12123038 | g.IVS12+2479G>C |
|  | rs3766735 | 1 | 12129011 | g.IVS12-900G>A |
|  | rs671106 | 1 | 12132445 | g.IVS13-107G>A |
|  | rs755398 | 1 | 12136361 | g.IVS14-249G>C |
|  | rs641941 | 1 | 12140918 | g.Ex15+2389A>G |
|  | rs631272 | 1 | 12141021 | g.Ex15+2492G>A |
|  | rs630542 | 1 | 12141132 | g.Ex15+2603G>T |
|  | rs816060 | 1 | 12154176 | g.-7238A>G |
|  | rs496888 | 1 | 12167072 | g.IVS1+5580T>C |
|  | rs976881 | 1 | 12168020 | g.IVS1+6528T>C |
|  | rs597519 | 1 | 12169151 | g.IVS1+7659T>C |
|  | rs5745984 | 1 | 12174681 | g.IVS1-8438C>G |
|  | rs816050 | 1 | 12176257 | g.IVS1-6862G>A |
|  | rs683240 | 1 | 12184095 | g.IVS2+877A>G |
|  | rs5746016 | 1 | 12185245 | g.IVS2-35C>T |
|  | rs590977 | 1 | 12189626 | g.IVS8+685A>C |
|  | rs235249 | 1 | 12192497 | g.IVS8+3556T>C |
|  | rs235219 | 1 | 12198621 | g.IVS9+2127A>G |

*Supplemental Table 2* (continued).

| **Region** | **DB SNP ID** | **Chromosome** | **Chromosomal position** | **Genomic position** |
| --- | --- | --- | --- | --- |
| *TNFRSF8/TNFRSF1B* (continued) | rs1061624 | 1 | 12201531 | g.Ex10+469A>G |
|  | rs1061631 | 1 | 12202765 | g.Ex10-779G>A |
|  | rs235214 | 1 | 12205769 | g.Ex10+2227T>C |
|  | rs4846100 | 1 | 12211930 | g.Ex2+3186G>C |
|  | rs7552664 | 1 | 12212233 | g.Ex2+2883G>T |
|  | rs7550488 | 1 | 12213198 | g.Ex2+1918T>C |
| *TNFRSF9* | rs664673 | 1 | 7904341 | g.*51464G>A |
|  | rs679563 | 1 | 7905344 | g.*48455C>T |
|  | rs226476 | 1 | 7933229 | g.IVS2-75A>C |
|  | rs2493215 | 1 | 7941982 | g.-42298T>C |
|  | rs226473 | 1 | 7943234 | g.-46054A>G |
| *TNFSF18* | rs6425201 | 1 | 169744461 | g.IVS2+275G>A |
|  | rs975074 | 1 | 169745424 | g.IVS1-658G>T |
|  | rs2236876 | 1 | 169749143 | g.IVS1+2395C>T |
|  | rs723858 | 1 | 169749920 | g.IVS1+1618A>T |
| *TNFSF4* | rs11811788 | 1 | 169882384 | g.Ex3+2145G>C |
|  | rs10489270 | 1 | 169889421 | g.IVS1-56C>T |
|  | rs7518045 | 1 | 169895169 | g.IVS1-5804T>C |
|  | rs10912560 | 1 | 169896114 | g.IVS1-6749T>C |
|  | rs1234313 | 1 | 169897904 | g.IVS1-8539T>C |
|  | rs3850641 | 1 | 169907489 | g.IVS1+331T>C |
|  | rs1234314 | 1 | 169909049 | g.Ex2G>C |
|  | rs1234315 | 1 | 169910120 | g.Ex2G>A |
|  | rs3861953 | 1 | 169912045 | g.Ex2G>A |
|  | rs1234317 | 1 | 169919432 | g.-57667G>A |
|  | rs2205960 | 1 | 169923132 | g.-68767C>A |
| *TRAF5* | rs7514863 | 1 | 207872495 | g.IVS9-1962A>T |
|  | rs12723208 | 1 | 207899289 | g.IVS1-8812A>G |
|  | rs6672742 | 1 | 207906160 | g.IVS1-1941A>G |
|  | rs6684874 | 1 | 207920455 | g.IVS5-1194A>T |
|  | rs3738199 | 1 | 207933739 | g.IVS11-126A>G |
|  | rs12569232 | 1 | 207941459 | g.Ex2G>C |
| *CFLAR* | rs2110728 | 2 | 201791184 | g.*15339G>A |
|  | rs7583529 | 2 | 201813744 | g.IVS1-6214G>A |
|  | rs12620435 | 2 | 201821285 | g.IVS2+910A>G |
| *REL* | rs842636 | 2 | 61003601 |  |
|  | rs842640 | 2 | 61007156 |  |
|  | rs13019590 | 2 | 61011071 |  |
|  | rs10203477 | 2 | 61016636 |  |
|  | rs7604989 | 2 | 61045444 | g.IVS4+5575A>G |
|  | rs1429265 | 2 | 61064733 | g.Ex11+3113G>T |
|  | rs3732179 | 2 | 61065976 | g.*4654T>C |
|  | rs11678166 | 2 | 61069371 | g.*110681C>T |
| *TANK* | rs2884364 | 2 | 161803154 |  |
|  | rs1267082 | 2 | 161805389 |  |
|  | rs4664406 | 2 | 161810654 |  |
|  | rs17705608 | 2 | 161816704 |  |
|  | rs7568498 | 2 | 161854620 | g.IVS1-7012T>G |
|  | rs1267072 | 2 | 161873088 | g.IVS2+11309C>T |
|  | rs1921310 | 2 | 161874708 | g.IVS2-10797A>G |
|  | rs1267033 | 2 | 161896845 | g.IVS4-9088T>C |
|  | rs1267034 | 2 | 161898245 | g.IVS4-7688A>G |
|  | rs7309 | 2 | 161918147 | g.Ex8-43G>A |
| *TNFSF10* | rs2041693 | 3 | 173698277 | g.*42290G>A |
|  | rs3136609 | 3 | 173708187 | g.IVS4-776C>T |
|  | rs3136607 | 3 | 173708476 | g.IVS4-1065T>A |
|  | rs3136597 | 3 | 173711246 | g.IVS3+863C>A |
|  | rs2241063 | 3 | 173712573 | g.IVS2-422C>A |
|  | rs3136594 | 3 | 173713286 | g.IVS2-1135C>T |
|  | rs4894559 | 3 | 173716071 | g.IVS1-581T>C |
|  | rs231983 | 3 | 173719142 | g.IVS1-3652A>C |
|  | rs2270418 | 3 | 173723701 | g.IVS1+44A>C |
|  | rs365238 | 3 | 173724568 | g.Ex2G>A |
|  | rs3136581 | 3 | 173725863 | g.-23574C>T |

*Supplemental Table 2* (continued).

| **Region** | **DB SNP ID** | **Chromosome** | **Chromosomal position** | **Genomic position** |
| --- | --- | --- | --- | --- |
| *TNFSF10 (continued)* | rs233998 | 3 | 173726448 | g.Ex2C>T |
|  | rs11918343 | 3 | 173730482 | g.-37431G>A |
|  | rs232006 | 3 | 173730847 | g.-38526G>A |
|  | rs3850170 | 3 | 173735004 | g.-50997G>A |
|  | rs9879554 | 3 | 173738107 | g.-60306T>A |
|  | rs17601879 | 3 | 173740833 | g.-68484A>G |
| *NFKB1* | rs3774933 | 4 | 103783524 | g.IVS1+3392T>C |
|  | rs3774934 | 4 | 103784661 | g.IVS1+4529A>G |
|  | rs4648006 | 4 | 103818743 | g.IVS5+2447C>T |
|  | rs230515 | 4 | 103828618 | g.IVS5+12322A>G |
|  | rs230510 | 4 | 103833356 | g.IVS5-11979A>T |
|  | rs4648022 | 4 | 103853630 | g.IVS6-1596C>T |
|  | rs1598859 | 4 | 103863637 | g.IVS11+467T>C |
|  | rs4648095 | 4 | 103885069 | g.IVS17+22T>C |
|  | rs4648110 | 4 | 103891022 | g.IVS22+58T>A |
|  | rs4648141 | 4 | 103894102 | g.IVS23-690G>A |
|  | rs7674640 | 4 | 103897983 | g.Ex24+2322C>T |
|  | rs997476 | 4 | 103899208 | g.Ex24+3547G>T |
|  | rs4698863 | 4 | 103903051 | g.*150455G>A |
| *IRF4* | rs9501958 | 6 | 323970 | g.-14182G>A |
|  | rs11756234 | 6 | 325328 | g.-12824G>A |
|  | rs1033180 | 6 | 328546 | g.-9606C>T |
|  | rs12203592 | 6 | 341321 | g.IVS4+386C>T |
|  | rs6900384 | 6 | 342937 | g.IVS5+685T>G |
|  | rs13208928 | 6 | 348470 | g.IVS7-1548T>A |
|  | rs3778607 | 6 | 348799 | g.IVS7-1219A>G |
|  | rs3800262 | 6 | 350900 | g.IVS8+770A>G |
|  | rs1050975 | 6 | 353012 | g.Ex9+558G>A |
|  | rs7768807 | 6 | 353246 | g.Ex9+792T>C |
|  | rs12211228 | 6 | 353833 | g.Ex9+1379G>C |
|  | rs11242865 | 6 | 356954 | g.Ex9+762C>T |
|  | rs7757906 | 6 | 357741 | g.Ex9+1549A>G |
|  | rs11242867 | 6 | 360406 | g.*7807A>G |
|  | rs9378805 | 6 | 362727 | g.*10128A>C |
| *LTA/TNF* | rs6916921 | 6 | 31628405 | g.IVS2+4210C>T |
|  | rs2857605 | 6 | 31632830 | g.IVS2-554C>T |
|  | rs2239707 | 6 | 31633298 | g.IVS2-86C>T |
|  | rs2230365 | 6 | 31633427 | g.Ex3+44C>T |
|  | rs3130062 | 6 | 31633891 | g.Ex4+114C>T |
|  | rs4947324 | 6 | 31636109 | g.*1741C>T |
|  | **rs1800629** | 6 | 31651010 |  |
|  | **rs361525** | 6 | 31651080 |  |
|  | **rs1799724** | 6 | 31650461 |  |
|  | **rs1800630** | 6 | 31650455 |  |
|  | **rs909253** | 6 | 31648292 |  |
|  | **rs2239704** | 6 | 31648120 |  |
|  | rs2844484 | 6 | 31644203 | g.-4295A>G |
|  | rs2844482 | 6 | 31647746 | g.-752C>T |
|  | rs2857713 | 6 | 31648535 | g.Ex2+46T>C |
|  | rs3093662 | 6 | 31652168 | g.IVS1-122A>G |
|  | rs2256974 | 6 | 31663371 | g.IVS3-26C>A |
| *NFKBIE* | rs13296 | 6 | 44326098 | g.Ex6+93A>G |
|  | rs1875324 | 6 | 44330988 | g.Ex4+372C>A |
|  | rs520639 | 6 | 44332969 | g.IVS1+146G>A |
|  | rs2282151 | 6 | 44334173 | g.*8321A>G |
|  | rs730775 | 6 | 44340052 | g.IVS1+645T>C |
|  | rs483536 | 6 | 44343831 | g.-14107A>T |
|  | rs1044690 | 6 | 44353219 | g.IVS4-266G>C |
|  | rs12664825 | 6 | 44360231 | g.IVS2+1448G>A |
|  | rs324140 | 6 | 44360606 | g.IVS2+1073G>A |
|  | rs9381307 | 6 | 44361369 | g.IVS2+310G>A |

*Supplemental Table 2* (continued).

| **Region** | **DB SNP ID** | **Chromosome** | **Chromosomal position** | **Genomic position** |
| --- | --- | --- | --- | --- |
| *IKBKB* | rs3747811 | 8 | 42248662 | g.IVS1-96A>T |
|  | rs11986055 | 8 | 42254335 | g.IVS2+5455A>C |
|  | rs5029748 | 8 | 42259706 | g.IVS2-5603G>T |
|  | rs4560769 | 8 | 42259961 | g.IVS2-5348A>G |
|  | rs2272733 | 8 | 42277059 | g.IVS5-4803T>C |
|  | rs10958713 | 8 | 42299873 | g.IVS19+704C>T |
|  | rs3763511 | 8 | 42355015 | g.IVS1+130C>T |
| *TNFRSF10B/TNFRSF10C/TNFRSF10D/TNFRSF10A* | rs2466183 | 8 | 22928002 | g.IVS7-146G>T |
|  | rs876435 | 8 | 22929478 | g.IVS9+277G>A |
|  | rs2889 | 8 | 22931854 | g.Ex10+1145A>G |
|  | rs1047275 | 8 | 22936107 | g.Ex9+336C>G |
|  | rs6557609 | 8 | 22937797 | g.IVS7-79A>G |
|  | rs883429 | 8 | 22942763 | g.IVS4+305G>A |
|  | rs4460370 | 8 | 22943553 | g.IVS3-374C>T |
|  | rs11785599 | 8 | 22948219 | g.IVS2-3889A>G |
|  | rs7834266 | 8 | 22954349 | g.IVS2+2247G>A |
|  | rs1001793 | 8 | 22956894 | g.IVS1-193G>A |
|  | rs12677679 | 8 | 22967018 | g.IVS1-10317T>C |
|  | rs11135693 | 8 | 22981099 | g.IVS1+1110T>G |
|  | rs4872049 | 8 | 22987724 | g.-64586A>G |
|  | rs4268128 | 8 | 22993801 | g.-82817C>T |
|  | rs9314259 | 8 | 22996545 | g.-91049T>C |
|  | rs9644029 | 8 | 23002369 | g.-14210A>G |
|  | rs4518666 | 8 | 23010150 | g.-6429T>C |
|  | rs11778416 | 8 | 23010250 | g.-6329C>G |
|  | rs4872052 | 8 | 23010877 | g.-5702T>C |
|  | rs4871846 | 8 | 23011252 | g.-5327C>G |
|  | rs12545733 | 8 | 23012948 | g.Ex2T>C |
|  | rs7008760 | 8 | 23013064 | g.Ex2G>C |
|  | rs4077341 | 8 | 23018293 | g.IVS1+1654T>G |
|  | rs12546235 | 8 | 23018464 | g.IVS1+1825C>T |
|  | rs11786012 | 8 | 23024330 | g.IVS1-848T>C |
|  | rs10111172 | 8 | 23025036 | g.IVS1-142T>C |
|  | rs10866820 | 8 | 23033115 | g.Ex5+2221A>G |
|  | rs4242390 | 8 | 23041344 | g.*53831G>A |
|  | rs7843320 | 8 | 23043782 | g.*46517G>A |
|  | rs4278155 | 8 | 23052724 | g.IVS7-1006T>G |
|  | rs4242391 | 8 | 23056128 | g.IVS7+1780A>G |
|  | rs6557618 | 8 | 23057070 | g.IVS7+838T>A |
|  | rs3924519 | 8 | 23060574 | g.IVS3-44A>G |
|  | rs4871850 | 8 | 23062121 | g.IVS2-112C>T |
|  | rs13257094 | 8 | 23063123 | g.IVS2-1114C>T |
|  | rs7014131 | 8 | 23064551 | g.IVS2-2542A>T |
|  | rs4872067 | 8 | 23076032 | g.IVS1+1212T>A |
|  | rs4871854 | 8 | 23097027 | g.*57570A>C |
|  | rs2230229 | 8 | 23105237 | g.Ex10+235G>A |
|  | rs11779484 | 8 | 23107300 | g.IVS9-1829A>G |
|  | rs11775256 | 8 | 23107430 | g.IVS9-1959G>A |
|  | rs11780345 | 8 | 23107911 | g.IVS9-2440A>G |
|  | rs3808530 | 8 | 23111674 | g.IVS8-1012T>C |
|  | rs6557627 | 8 | 23112116 | g.IVS8+608G>C |
|  | rs2235126 | 8 | 23113126 | g.IVS7+218G>A |
|  | rs4242392 | 8 | 23117578 | g.IVS2-1359A>G |
|  | rs6995408 | 8 | 23117823 | g.IVS2-1604C>T |
|  | rs7820465 | 8 | 23129631 | g.IVS1-3961C>T |
|  | rs6557638 | 8 | 23129747 | g.IVS1-4077G>C |
|  | rs13278062 | 8 | 23138916 | g.-34823C>A |
|  | **rs20577** | 8 | 23138422 |  |
|  | rs4242394 | 8 | 23144190 | g.Ex3-195T>C |
|  | rs11777697 | 8 | 23144929 | g.Ex3+546C>G |
|  | rs11997323 | 8 | 23150340 | g.*6911A>C |
|  | rs7463256 | 8 | 23157565 | g.Ex2T>C |
|  | rs11135710 | 8 | 23158030 | g.Ex2T>A |

*Supplemental Table 2* (continued).

| **Region** | **DB SNP ID** | **Chromosome** | **Chromosomal position** | **Genomic position** |
| --- | --- | --- | --- | --- |
| *TNFSF8* | rs1322055 | 9 | 114749139 | g.IVS2-1406G>A |
|  | rs3789882 | 9 | 114749253 | g.IVS2-1520T>A |
|  | rs10982448 | 9 | 114751798 | g.IVS2-4065A>G |
|  | rs1555457 | 9 | 114753874 | g.IVS2+6056G>A |
|  | rs7872878 | 9 | 114761631 | g.IVS1-1659C>G |
|  | rs3181360 | 9 | 114771112 | g.IVS1+831G>A |
|  | rs4979474 | 9 | 114775538 | g.Ex2C>T |
|  | rs2181033 | 9 | 114777385 | g.-43015T>C |
|  | rs927375 | 9 | 114777501 | g.-43363C>T |
|  | rs17292115 | 9 | 114779299 | g.-48757T>G |
|  | rs10817686 | 9 | 114779404 | g.-49072A>G |
|  | rs4979476 | 9 | 114780264 | g.-51652C>T |
|  | rs10982461 | 9 | 114780962 | g.-53746G>T |
|  | rs10759743 | 9 | 114781306 | g.-54778T>C |
|  | rs11791315 | 9 | 114783709 | g.-61987C>A |
| *TRAF2* | rs2784081 | 9 | 137067728 | g.-1301C>A |
|  | rs4880073 | 9 | 137093190 | g.IVS8-951G>A |
|  | rs908831 | 9 | 137098379 | g.*2188C>T |
|  | rs4880166 | 9 | 137102410 | g.*6219T>G |
| *CHUK* | rs7903344 | 10 | 101967873 | g.Ex9+5G>A |
|  | rs11595324 | 10 | 101969408 | g.IVS5-302A>G |
|  | rs12764370 | 10 | 101969472 | g.IVS5-366A>G |
|  | rs17668357 | 10 | 101993896 | g.IVS9-372C>G |
| *FAS* | rs17447091 | 10 | 90721249 | g.-19364T>A |
|  | rs11202916 | 10 | 90726379 | g.-14234A>G |
|  | rs17447140 | 10 | 90728927 | g.-11686A>T |
|  | rs12765241 | 10 | 90730481 | g.-10132G>A |
|  | rs4934433 | 10 | 90730933 | g.-9680C>A |
|  | rs2862834 | 10 | 90731354 | g.-9259A>G |
|  | rs10887876 | 10 | 90735110 | g.-5503C>T |
|  | rs4934434 | 10 | 90737149 | g.Ex2C>A |
|  | rs3758483 | 10 | 90738716 | g.Ex2C>T |
|  | rs10509561 | 10 | 90741892 | g.IVS1+1249T>A |
|  | rs6586165 | 10 | 90745036 | g.IVS1+4393T>A |
|  | rs1571011 | 10 | 90747767 | g.IVS1-4999A>C |
|  | rs2147420 | 10 | 90749593 | g.IVS1-3173A>G |
|  | rs1159120 | 10 | 90749672 | g.IVS1-3094C>T |
|  | rs9658727 | 10 | 90751845 | g.IVS1-921T>C |
|  | rs7901656 | 10 | 90756193 | g.IVS2-1244T>C |
|  | rs7911226 | 10 | 90758945 | g.IVS4+211A>G |
|  | rs9658761 | 10 | 90759866 | g.IVS4-410G>T |
|  | rs2234978 | 10 | 90761809 | g.Ex7-10T>C |
|  | rs1051070 | 10 | 90764752 | g.Ex9-771A>T |
|  | rs12257092 | 10 | 90772807 | g.*8619A>T |
|  | rs10887883 | 10 | 90772953 | g.*8765A>G |
|  | rs4934436 | 10 | 90773300 | g.*9112C>T |
|  | **rs1800682** | 10 | 90739943 |  |
|  | **rs1468063** | 10 | 90765271 |  |
| *NFKB2* | rs1057050 | 10 | 104132284 | g.Ex40-356G>A |
|  | rs11191275 | 10 | 104132790 | g.Ex40+152C>T |
|  | rs7076748 | 10 | 104140132 | g.-5574G>C |
|  | rs11574849 | 10 | 104149686 | g.IVS14-141G>A |
|  | rs1056890 | 10 | 104152760 | g.Ex18-395C>T |
| *FADD* | rs7930377 | 11 | 69710476 | g.IVS24-1016G>A |
|  | rs481845 | 11 | 69711231 | g.IVS24-261A>G |
|  | rs10898840 | 11 | 69717569 | g.*5810C>T |
|  | rs7939734 | 11 | 69732593 | g.Ex2+1450T>A |
|  | rs10751209 | 11 | 69736753 | g.*6525C>T |
| *NFRKB* | rs3087920 | 11 | 129234077 | g.Ex6+399C>G |
|  | rs1622182 | 11 | 129235305 | g.Ex6+198T>C |
|  | rs951838 | 11 | 129236893 | g.*3102G>A |
|  | rs1650825 | 11 | 129248603 | g.IVS20+247T>C |
|  | rs1016364 | 11 | 129250082 | g.IVS16-61T>C |

*Supplemental Table 2* (continued)

| **Region** | **DB SNP ID** | **Chromosome** | **Chromosomal position** | **Genomic position** |
| --- | --- | --- | --- | --- |
| *NFRKB* (continued) | rs1733448 | 11 | 129253947 | g.IVS11-299A>G |
|  | rs11820190 | 11 | 129256367 | g.IVS11+181A>G |
|  | rs2293664 | 11 | 129258201 | g.IVS7+26C>G |
|  | rs3829264 | 11 | 129269778 | g.Ex2G>A |
|  | rs1650817 | 11 | 129278734 | g.IVS21-1113G>T |
|  | rs1161662 | 11 | 129284897 | g.IVS20+684G>A |
|  | rs1650815 | 11 | 129285178 | g.IVS20+403G>A |
|  | rs3734075 | 11 | 129287006 | g.IVS19+184G>A |
| *RELA* | rs2448490 | 11 | 65168611 | g.IVS5-391G>A |
|  | rs1466462 | 11 | 65175940 | g.*1177G>C |
|  | rs11227247 | 11 | 65179429 | g.IVS10+306T>G |
|  | rs11820062 | 11 | 65186512 | g.IVS1-260A>G |
| *TRAF6* | rs331457 | 11 | 36480188 | g.IVS1-251G>A |
|  | rs5030411 | 11 | 36490329 | g.Ex2C>T |
|  | rs331455 | 11 | 36497125 | g.-56036G>A |
|  | rs11033695 | 11 | 36541051 | g.-10379A>T |
|  | rs2227973 | 11 | 36553889 | g.Ex2+2473A>G |
|  | rs12421641 | 11 | 36562795 | g.*8232C>A |
|  | rs1818545 | 11 | 36568666 | g.Ex2+1406G>A |
|  | rs10768219 | 11 | 36579047 | g.IVS1+6329C>T |
| *TNFRSF1A/LTBR/TNFRSF7* | rs1860545 | 12 | 6317038 | g.IVS1-3367C>T |
|  | rs4149579 | 12 | 6317618 | g.IVS1+3585G>A |
|  | rs4149578 | 12 | 6317698 | g.IVS1+3505G>A |
|  | rs4149577 | 12 | 6317783 | g.IVS1+3420C>T |
|  | rs4149570 | 12 | 6321851 | g.Ex2T>G |
|  | rs11064145 | 12 | 6325359 | g.*32474A>C |
|  | rs3764875 | 12 | 6328345 | g.IVS11+32G>A |
|  | rs3764874 | 12 | 6328535 | g.IVS10+56C>G |
|  | rs3782723 | 12 | 6336316 | g.IVS4-1009C>G |
|  | rs3782726 | 12 | 6339932 | g.IVS4+1546C>A |
|  | rs10849446 | 12 | 6349553 | g.IVS2+4242T>G |
|  | rs3759324 | 12 | 6355922 | g.Ex2A>G |
|  | rs3759333 | 12 | 6362208 | g.Ex2C>T |
|  | rs2364480 | 12 | 6365536 | g.Ex5+44C>A |
|  | rs12296430 | 12 | 6373761 | g.Ex10+2767G>C |
|  | rs12319859 | 12 | 6409752 | g.-8176T>C |
|  | rs2364493 | 12 | 6415792 | g.Ex2A>G |
|  | rs4469949 | 12 | 6415872 | g.Ex2G>A |
|  | rs12817967 | 12 | 6416255 | g.Ex2A>C |
|  | rs7312492 | 12 | 6420012 | g.IVS1-933T>C |
|  | rs2250246 | 12 | 6423277 | g.-1245A>G |
|  | rs11569357 | 12 | 6423597 | g.-925T>C |
|  | rs11569359 | 12 | 6423865 | g.-657C>G |
|  | rs25680 | 12 | 6424889 | g.Ex2+39G>A |
|  | rs2267966 | 12 | 6427453 | g.IVS2-2147A>T |
|  | rs2286721 | 12 | 6432326 | g.IVS1-168G>A |
|  | rs2532499 | 12 | 6433516 | g.IVS3+373C>G |
|  | rs2243750 | 12 | 6438048 | g.IVS4-24T>C |
|  | rs1045546 | 12 | 6438168 | g.Ex5+97C>T |
|  | rs2534711 | 12 | 6441082 | g.IVS6-379T>G |
| *TNFSF13B* | rs915047 | 13 | 107648692 | g.*36483C>T |
|  | rs9514825 | 13 | 107650277 | g.*31728A>G |
|  | rs17497319 | 13 | 107651063 | g.*29370A>G |
|  | rs1105451 | 13 | 107651324 | g.*28587C>T |
|  | rs868284 | 13 | 107652214 | g.*25917C>T |
|  | rs1555902 | 13 | 107654784 | g.Ex3+3012G>T |
|  | rs1151403 | 13 | 107656374 | g.Ex3+1422G>A |
|  | rs10131 | 13 | 107657847 | g.Ex3-53G>A |
|  | rs9520823 | 13 | 107671565 | g.-29399A>C |
|  | rs1473792 | 13 | 107675630 | g.-41594A>G |
|  | rs11619378 | 13 | 107679090 | g.Ex1T>G |
|  | rs12869406 | 13 | 107684951 | g.Ex1+2615A>T |
|  | rs1224096 | 13 | 107701073 | g.-19171G>A |
|  | rs2582869 | 13 | 107710146 | g.-10098A>G |

*Supplemental Table 2* (continued)

| **Region** | **DB SNP ID** | **Chromosome** | **Chromosomal position** | **Genomic position** |
| --- | --- | --- | --- | --- |
| *TNFSF13B* | rs1224177 | 13 | 107711357 | g.-8887G>T |
|  | rs9587544 | 13 | 107712281 | g.-7963G>A |
|  | rs17498722 | 13 | 107713986 | g.-6258G>A |
|  | rs1041569 | 13 | 107717544 | g.Ex2T>A |
|  | rs9514828 | 13 | 107719374 | g.Ex2C>T |
|  | rs8181791 | 13 | 107732046 | g.IVS2-5106G>A |
|  | rs16972216 | 13 | 107732621 | g.IVS2-4531G>A |
|  | rs17499386 | 13 | 107735314 | g.IVS2-1838T>C |
|  | rs12583006 | 13 | 107735453 | g.IVS2-1699T>A |
|  | rs10508198 | 13 | 107740789 | g.IVS3+3581G>C |
|  | rs9520836 | 13 | 107755064 | g.IVS5+1111A>G |
|  | rs4145212 | 13 | 107758189 | g.Ex6+824T>A |
|  | rs1224163 | 13 | 107763060 | g.*5772T>G |
|  | rs16972257 | 13 | 107764642 | g.*7354T>C |
|  | rs1224166 | 13 | 107765412 | g.*8124C>T |
| *NFKBIA* | rs7152826 | 14 | 34932155 | g.*28667G>T |
|  | rs3138056 | 14 | 34938265 | g.Ex6+2204G>A |
|  | rs3138045 | 14 | 34947472 | g.Ex2A>G |
|  | rs2007960 | 14 | 34952686 | g.-30293T>A |
|  | rs17103274 | 14 | 34953429 | g.-32522A>G |
|  | rs17103282 | 14 | 34954566 | g.-35933G>A |
|  | rs17103286 | 14 | 34954792 | g.-36611T>C |
|  | rs8018407 | 14 | 34955037 | g.-37346A>G |
|  | rs8018193 | 14 | 34955498 | g.-38729C>T |
|  | rs2415290 | 14 | 34956706 | g.NC_-12001C>T |
|  | rs8008601 | 14 | 34957182 | g.NC_-11525C>T |
|  | rs762009 | 14 | 34960612 | g.NC_-8095A>G |
|  | rs4982271 | 14 | 34963376 | g.NC_-5331G>T |
| *TNFRSF12A* | rs1859378 | 16 | 2991124 |  |
|  | rs11862306 | 16 | 3001929 | g.-1435T>C |
|  | rs2227269 | 16 | 3003265 | g.Ex2+447C>T |
|  | rs2717701 | 16 | 3006169 | g.IVS1-125C>G |
|  | rs8052002 | 16 | 3010789 | g.IVS1+296G>C |
|  | rs2245000 | 16 | 3016000 | g.IVS3+10C>G |
| *TNFRSF17* | rs13330634 | 16 | 11948012 |  |
|  | rs9928563 | 16 | 11949370 |  |
|  | rs11862958 | 16 | 11962692 | g.Ex2C>T |
|  | rs11570151 | 16 | 11968080 | g.IVS2+381T>C |
|  | rs2071336 | 16 | 11969175 | g.Ex3+248G>A |
|  | rs12597429 | 16 | 11969897 | g.Ex3+472T>C |
|  | rs3851005 | 16 | 11978714 | g.IVS1+543C>T |
|  | rs2869492 | 16 | 11978842 | g.IVS1+671T>C |
| *TRADD* | rs7184692 | 16 | 65737672 | g.IVS14-10T>C |
|  | rs9033 | 16 | 65739500 | g.Ex16-444A>G |
|  | rs13312735 | 16 | 65744712 | g.*8827C>A |
|  | rs2233455 | 16 | 65765434 | g.Ex1-17C>T |
|  | rs8057598 | 16 | 65766756 | g.Ex4+306A>G |
| *TNFRSF13B* | rs3751991 | 17 | 16776011 | g.IVS5+199G>T |
|  | rs11650590 | 17 | 16777025 | g.IVS2-49G>A |
|  | rs3751987 | 17 | 16777150 | g.Ex2-47C>T |
|  | rs8065836 | 17 | 16778090 | g.Ex2+481T>C |
|  | rs9904659 | 17 | 16778369 | g.Ex2+202A>G |
|  | rs4343329 | 17 | 16781614 | g.*37994C>G |
|  | rs11654431 | 17 | 16785588 | g.IVS3-1038G>T |
|  | rs12938073 | 17 | 16786901 | g.IVS3-2351A>T |
|  | rs12603708 | 17 | 16787838 | g.IVS3-3288C>T |
|  | rs11654088 | 17 | 16790538 | g.IVS3+2239C>G |
|  | rs4985694 | 17 | 16802057 | g.IVS1-5435C>T |
|  | rs6502541 | 17 | 16802955 | g.IVS1-6333G>C |
|  | rs12051889 | 17 | 16803897 | g.IVS1-7275G>A |
|  | rs4985726 | 17 | 16804363 | g.IVS1-7741G>C |
|  | rs4985700 | 17 | 16806800 | g.IVS1+9254G>T |
|  | rs7504096 | 17 | 16817808 | g.Ex2T>C |
|  | rs8074984 | 17 | 16819795 | g.Ex2C>T |

*Supplemental Table 2* (continued)

| **Region** | **DB SNP ID** | **Chromosome** | **Chromosomal position** | **Genomic position** |
| --- | --- | --- | --- | --- |
| *TNFRSF13B* (continued) | rs4985757 | 17 | 16820148 | g.-45078T>G |
|  | rs8064661 | 17 | 16823389 | g.-54801A>G |
|  | rs8079130 | 17 | 16825685 | g.-61689A>G |
|  | rs12943647 | 17 | 16826611 | g.-64467C>T |
|  | rs9891079 | 17 | 16830946 | g.-77472T>G |
|  | rs4075606 | 17 | 16831544 | g.-79266A>G |
|  | rs12950509 | 17 | 16832671 | g.-82647G>C |
|  | rs9907308 | 17 | 16832990 | g.-83604C>T |
|  | rs4985767 | 17 | 16835885 | g.-92289T>C |
| *TNFSF12* | rs4968200 | 17 | 7389181 | g.-4013C>G |
|  | rs4968212 | 17 | 7408940 | g.IVS3+35T>C |
|  | rs10438740 | 17 | 7413608 | g.IVS7-790G>A |
| *RELB* | rs204468 | 19 | 50182476 | g.Ex8-46T>C |
|  | rs3859419 | 19 | 50184973 | g.IVS9-520T>G |
|  | rs875255 | 19 | 50185475 | g.IVS9-18C>G |
|  | rs6509177 | 19 | 50221846 | g.IVS7+1011A>G |
|  | rs10424046 | 19 | 50227876 | g.IVS9+29C>G |
|  | rs1560725 | 19 | 50235627 | g.IVS2+218C>T |
| *TNFSF14* | rs2279627 | 19 | 6614594 | g.Ex5+975C>G |
|  | rs1077667 | 19 | 6619972 | g.IVS2+890G>A |
|  | rs8106574 | 19 | 6622142 | g.Ex2A>G |
|  | rs379527 | 19 | 6627442 | g.Ex41+1437G>T |
|  | rs344555 | 19 | 6630360 | g.IVS37+58A>G |
|  | rs2277984 | 19 | 6630511 | g.IVS36-4G>A |
|  | rs344550 | 19 | 6633953 | g.IVS33-713G>C |
|  | rs1389623 | 19 | 6635197 | g.IVS33+202C>T |
|  | rs2241393 | 19 | 6636304 | g.IVS29-147C>G |
|  | rs344548 | 19 | 6636817 | g.IVS29+318C>G |
|  | rs237554 | 19 | 6637659 | g.IVS28+98A>G |
|  | rs344542 | 19 | 6638517 | g.IVS27-604T>C |
|  | rs11569523 | 19 | 6640042 | g.IVS27+1598G>A |
|  | rs11569515 | 19 | 6641244 | g.IVS27+396A>C |
| *TNFSF7* | rs12977027 | 19 | 6530789 | g.*23677C>T |
|  | rs2910434 | 19 | 6532596 | g.*18256C>A |
|  | rs1808398 | 19 | 6536298 | g.*7150G>A |
|  | rs16994592 | 19 | 6537498 | g.IVS2-82A>G |
|  | rs344586 | 19 | 6539231 | g.IVS2-1815G>C |
|  | rs344589 | 19 | 6541789 | g.IVS1+63T>C |
|  | rs17703895 | 19 | 6542146 | g.Ex1+18G>T |
|  | rs344591 | 19 | 6542489 | g.-6439G>A |
|  | rs344596 | 19 | 6545274 |  |
|  | rs168259 | 19 | 6546779 |  |
|  | rs344584 | 19 | 6555018 |  |
|  | rs189368 | 19 | 6558409 |  |
|  | rs344580 | 19 | 6560390 | g.-60142T>C |
|  | rs4807089 | 19 | 6561365 | g.-63067A>G |
| *TNFSF9* | rs4807874 | 19 | 6467492 | g.-50591C>T |
|  | rs10415368 | 19 | 6467594 | g.-50897A>G |
|  | rs238767 | 19 | 6468122 | g.-51508C>T |
|  | rs11085177 | 19 | 6471490 | g.-10557G>A |
|  | rs12151125 | 19 | 6475406 | g.-6641G>T |
|  | rs348337 | 19 | 6481656 | g.Ex2T>C |
|  | rs348389 | 19 | 6486637 | g.Ex3-297C>T |
|  | rs348373 | 19 | 6494342 | g.*8264T>C |
| *CD40* | rs6131012 | 20 | 44162068 | g.NC_-154A>G |
|  | rs6032661 | 20 | 44167218 | g.NC_*4996A>G |
|  | rs6065926 | 20 | 44169261 | g.-11128A>G |
|  | rs6074021 | 20 | 44170203 | g.-10186C>G |
|  | rs6074021 | 20 | 44170203 | g.-10186C>G |
|  | rs6131014 | 20 | 44170790 | g.-9599T>C |
|  | rs1009373 | 20 | 44175408 | g.-4981G>T |
|  | rs11569309 | 20 | 44181957 | g.IVS1+1517T>C |
|  | rs11569317 | 20 | 44184257 | g.IVS2-22C>G |
|  | rs3746821 | 20 | 44188518 | g.IVS5-168G>T |
|  | rs3765457 | 20 | 44190620 | g.IVS8+216A>G |

*Supplemental Table 2* (continued)

| **Region** | **DB SNP ID** | **Chromosome** | **Chromosomal position** | **Genomic position** |
| --- | --- | --- | --- | --- |
| *CD40* (continued) | rs3765459 | 20 | 44190814 | g.IVS8-114G>A |
|  | rs1883838 | 20 | 44195303 | g.Ex9+3965T>C |
|  | rs4813001 | 20 | 44196264 | g.*5177G>C |
|  | rs1535043 | 20 | 44201131 | g.*10044T>A |
| *TNFRSF13C* | rs5996088 | 22 | 40648413 | g.Ex2G>A |
|  | rs5758511 | 22 | 40660672 | g.IVS5-972C>T |
|  | rs6002548 | 22 | 40661540 | g.IVS5-1840A>C |
|  | rs6002551 | 22 | 40662652 | g.IVS5+1462C>T |
