# Supplementary material for: Common Gene Variants in the Tumor Necrosis Factor (TNF) and TNF Receptor Superfamilies and NF-kB Transcription Factors and Non-Hodgkin Lymphoma Risk
Source: PLoS One. 2009 Apr 24;4(4):e5360. doi: 10.1371/journal.pone.0005360 (PMC2669130; doi:10.1371/journal.pone.0005360)
Supplement: Table S3 — Supplemental Table 3 (0.10 MB DOC) [file pone.0005360.s003.doc]

Supplemental Table 3. Study-specific and pooled demographic and pathology characteristics of study participants in the NCI-SEER, Connecticut, and New South Wales (NSW) NHL case-control studies included in the present analysis.

|  | **NCI-SEER** | | **Connecticut** | | **NSW** | | **Pooled** | |
| --- | --- | --- | --- | --- | --- | --- | --- | --- |
|  | **Control** | **Case** | **Control** | **Case** | **Control** | **Case** | **Control** | **Case** |
|  | **n=828** | **n=990** | **n=515** | **n=436** | **n=465** | **n=520** | **n=1808** | **n=1946** |
|  | **N (%)** | **N (%)** | **N (%)** | **N (%)** | **N (%)** | **N (%)** | **N (%)** | **N (%)** |
| **Sex** |  |  |  |  |  |  |  |  |
| Male | 443 (53) | 536 (54) | - | - | 268 (58) | 304 (58) | 711 (39) | 840 (43) |
| Female | 385 (47) | 454 (46) | 515 (100) | 436 (100) | 197 (42) | 216 (42) | 1097 (61) | 1106 (57) |
|  |  |  |  |  |  |  |  |  |
| **Age (years)** |  |  |  |  |  |  |  |  |
| < 50 | 203 (25) | 277 (28) | 98 (19) | 86 (20) | 107 (23) | 121 (23) | 408 (23) | 484 (25) |
| 50-59 | 177 (21) | 235 (24) | 97 (19) | 89 (20) | 135 (29) | 171 (33) | 409 (23) | 495 (25) |
| 60-69 | 285 (34) | 311 (31) | 120 (23) | 110 (25) | 151 (32) | 154 (30) | 556 (31) | 575 (30) |
| 70+ | 163 (20) | 167 (17) | 200 (39) | 151 (35) | 72 (16) | 74 (14) | 435 (24) | 392 (20) |
|  |  |  |  |  |  |  |  |  |
| **Race** |  |  |  |  |  |  |  |  |
| White | 669 (81) | 858 (87) | 484 (94) | 420 (96) | 459 (99) | 507 (98) | 1612 (89) | 1785 (92) |
| Black | 112 (13) | 64 (6) | 14 (3) | 13 (3) | - | - | 126 (7) | 77 (4) |
| Asian | 16 (2) | 32 (3) | 3 (0.6) | 1 (0.2) | 6 (1) | 13 (2) | 25 (1) | 46 (2) |
| Other/Unknown | 31 (4) | 36 (4) | 14 (3) | 2 (0.5) | - | - | 45 (3) | 38 (2) |
|  |  |  |  |  |  |  |  |  |
| **Ethnicity** |  |  |  |  |  |  |  |  |
| Hispanic | 41 (5) | 52 (5) | 18 (4) | 6 (1) | - | - | 59 (3) | 58 (3) |
| Non-Hispanic | 787 (95) | 936 (95) | 491 (95) | 427 (98) | 465 (100) | 520 (100) | 1743 (96) | 1883 (97) |
| Unknown |  | 2 (0.2) | 6 (1) | 3 (1) |  |  | 6 (0.3) | 5 (0.3) |
|  |  |  |  |  |  |  |  |  |
| **Study Site** |  |  |  |  |  |  |  |  |
| Detroit | 139 (17) | 197 (20) | - | - | - | - | 139 (8) | 197 (10) |
| Iowa | 246 (30) | 301 (30) | - | - | - | - | 246 (14) | 301 (16) |
| L.A. | 199 (24) | 234 (24) | - | - | - | - | 199 (11) | 234 (12) |
| Seattle | 244 (29) | 258 (26) | - | - | - | - | 244 (13) | 258 (13) |
| Connecticut | - | - | 515 (100) | 436 (100) | - | - | 515 (28) | 436 (22) |
| N.S.W. | - | - | - | - | 446 (96) | 496 (95) | 446 (25) | 496 (26) |
| A.C.T. | - | - | - | - | 19 (4) | 24 (5) | 19 (1) | 24 (1) |
|  |  |  |  |  |  |  |  |  |
| **NHL Subtype** |  |  |  |  |  |  |  |  |
| DLBCL | - | 294 (30) | - | 137 (31) | - | 169 (33) | - | 600 (31) |
| Follicular | - | 246 (25) | - | 103 (24) | - | 191 (37) | - | 540 (28) |
| CLL/SLL | - | 101 (10) | - | 43 (10) | - | 17 (3) | - | 161 (8) |
| Mantle Cell | - | 40 (4) | - | 10 (2) | - | 19 (4) | - | 69 (4) |
| Marginal Zone | - | 82 (8) | - | 29 (7) | - | 49 (9) | - | 160 (8) |
| LPL | - | 24 (2) | - | 9 (2) | - | 23 (4) | - | 56 (3) |
| MF/SS | - | 18 (2) | - | 10 (2) | - | 3 (1) | - | 31 (2) |
| Burkitt | - | 11 (1) | - | 0 | - | 3 (1) | - | 14 (1) |
| Peripheral T | - | 41 (4) | - | 14 (3) | - | 7 (1) | - | 62 (3) |
| NOS | - | 133 (13) | - | 81 (19) | - | 39 (7) | - | 253 (13) |
|  |  |  |  |  |  |  |  |  |
| **DNA Source** |  |  |  |  |  |  |  |  |
| Blood | 598 (72) | 688 (70) | 515 (100) | 436 (100) | 465 (100) | 520 (100) | 1578 (87) | 1644 (85) |
| Buccal | 230 (28) | 302 (30) | - | - | - | - | 230 (13) | 302 (15) |

*Abbreviations:* NCI-SEER (National Cancer Institute-Surveillance Epidemiology and End Results), L.A. (Los Angeles), N.S.W. (New South Wales), A.C.T. (Australian Capital Territory), DLBCL (diffuse large B-cell lymphoma), CLL/SLL (chronic lymphocytic leukemia/small lymphocytic lymphoma), LPL (lymphoplasmacytic lymphoma), MF/SS (mycosis fungoides/sezary syndrome), NOS (not otherwise specified)
