# Supplementary material for: Common Gene Variants in the Tumor Necrosis Factor (TNF) and TNF Receptor Superfamilies and NF-kB Transcription Factors and Non-Hodgkin Lymphoma Risk
Source: PLoS One. 2009 Apr 24;4(4):e5360. doi: 10.1371/journal.pone.0005360 (PMC2669130; doi:10.1371/journal.pone.0005360)
Supplement: Table S4 — Supplemental Table 4 (0.11 MB DOC) [file pone.0005360.s004.doc]

Supplemental Table 4. Significance levels (p values) for the TNF/NFkB pathway and for each target region calculated calculated from the (a) permutation test [based on 10,000 permutations and the minimum p-trend within each region], and (b) likelihood ratio test [based on p-trends within each region], for the association with NHL and NHL subtypes (DLBCL, follicular, marginal zone, and CLL/SLL).

|  | **NHL** |  | **DLBCL** |  | **Follicular** |  | **CLL/SLL** |  | **Marginal Zone** |  |
| --- | --- | --- | --- | --- | --- | --- | --- | --- | --- | --- |
|  | **Permutation p** | **LRT** | **Permutation p** | **LRT** | **Permutation p** | **LRT** | **Permutation p** | **LRT** | **Permutation p** | **LRT** |
| TNF/NFkB pathway (tail strength) | **0.022** |  | 0.065 |  | 0.101 |  | 0.132 |  | 0.081 |  |
| ***By region*** |  |  |  |  |  |  |  |  |  |  |
| *CD40* | 0.3659 | 0.0861 | 0.3918 | 0.4330 | 0.5233 | 0.5004 | 0.4012 | 0.2607 | **0.0464** | 0.1571 |
| *CFLAR* | 0.0761 | 0.1776 | 0.1326 | 0.2738 | **0.0492** | 0.1351 | 0.7806 | 0.3741 | 0.7170 | 0.3052 |
| *CHUK* | 0.9221 | 0.7821 | 0.8594 | 0.8329 | 0.8541 | 0.4829 | 0.0938 | 0.4554 | 0.7208 | 0.7088 |
| *FADD* | 0.3490 | - | 0.4869 | - | 0.3493 | - | 0.9676 | - | 0.4180 | - |
| *FAS* | **0.0455** | **0.0465** | 0.1100 | 0.0904 | **0.0063** | **0.0225** | 0.4381 | 0.3488 | 0.2970 | **0.0380** |
| *FASLG* | 0.6791 | - | 0.4564 | - | 0.7885 | - | 0.8983 | - | 0.9284 | - |
| *IKBKB* | 0.1422 | 0.5730 | 0.1095 | 0.1798 | 0.3401 | 0.5375 | 0.3401 | 0.7635 | 0.8728 | 0.9202 |
| *IRF4* | **0.0319** | 0.6023 | 0.0942 | 0.3758 | 0.6168 | 0.9314 | 0.2028 | 0.3897 | 0.3170 | 0.1795 |
| *TNFSF13B* | **0.0355** | **0.0067** | **0.0475** | 0.1598 | 0.1605 | 0.0558 | **0.0012** | **0.0097** | 0.7140 | 0.4163 |
| *LTA/TNF* | 0.0736 | **0.0079** | **0.0214** | **0.0025** | 0.0718 | **0.0270** | 0.8966 | 0.9258 | 0.1379 | **0.0162** |
| *NFKB1* | 0.9127 | - | 0.7146 | - | 0.7940 | - | 0.9648 | - | 0.8298 | - |
| *NFKB2* | 0.0961 | 0.3282 | 0.5473 | 0.6666 | 0.1538 | 0.1773 | 0.9743 | 0.8832 | 0.1085 | 0.2703 |
| *NFKBIA* | 0.7776 | 0.6656 | 0.1062 | 0.0554 | 0.6729 | 0.4702 | 0.4511 | 0.5010 | 0.9125 | 0.5638 |
| *NFKBIE* | 0.0890 | **0.0358** | 0.2632 | 0.2216 | 0.3034 | 0.4829 | 0.2082 | 0.3876 | 0.1639 | 0.1483 |
| *NFRKB* | 0.8042 | 0.3306 | 0.3566 | 0.5411 | 0.6748 | 0.2273 | 0.2835 | 0.2078 | 0.7453 | 0.5683 |
| *REL* | 0.2956 | - | 0.4892 | - | 0.9294 | - | 0.3730 | - | 0.3850 | - |
| *RELA* | 0.2516 | 0.1814 | 0.6720 | 0.6405 | 0.4327 | 0.2602 | 0.3406 | 0.4763 | 0.1619 | 0.3032 |
| *RELB* | 0.5511 | 0.5687 | 0.1923 | 0.1693 | 0.6026 | 0.7879 | 0.0880 | **0.0260** | 0.4856 | 0.5715 |
| *TANK* | **0.0214** | 0.1529 | 0.4463 | 0.2954 | 0.2271 | 0.3574 | 0.1920 | 0.2427 | 0.6787 | 0.4641 |
| *TNFRSF10B/TNFRSF10C/TNFRSF10D/TNFRSF10A* | 0.7328 | 0.2497 | 0.4175 | 0.1528 | 0.9296 | 0.1197 | 0.5718 | 0.1119 | 0.5053 | 0.4095 |
| *TNFRSF12A* | 0.9257 | - | 0.9791 | - | 0.5988 | - | 0.4190 | - | 0.2273 | - |
| *TNFRSF13B* | 0.7047 | 0.8587 | 0.6549 | 0.6692 | 0.8725 | 0.9962 | 0.4310 | 0.2679 | 0.0717 | 0.1347 |
| *TNFRSF13C* | **0.0265** | **0.0488** | 0.5339 | 0.4422 | 0.1121 | 0.2067 | 0.2626 | 0.3252 | **0.0446** | **0.0066** |
| *TNFRSF14* | 0.4016 | - | 0.4512 | - | 0.1679 | - | 0.8420 | - | 0.3299 | - |
| *TNFRSF17* | 0.8780 | - | 0.9055 | - | 0.8848 | - | 0.3840 | - | 0.5099 | - |
| *TNFRSF1A/LTBR/*  *TNFRSF7* | 0.1535 | 0.0513 | 0.4633 | 0.3107 | 0.4300 | 0.1218 | 0.4978 | 0.1617 | 0.0820 | 0.0633 |
| *TNFRSF25* | 0.1234 | 0.1331 | 0.5634 | 0.6711 | **0.0397** | **0.0477** | 0.2674 | 0.2073 | 0.3662 | 0.5598 |
| *TNFRSF8/TNFRSF1B* | 0.7882 | 0.4563 | 0.3061 | 0.5632 | 0.3143 | 0.3949 | 0.3210 | 0.2802 | **0.0385** | 0.5681 |
| *TNFRSF9* | 0.3387 | 0.4545 | 0.1221 | 0.1949 | 0.5622 | 0.6953 | 0.5615 | 0.1554 | 0.5566 | 0.5877 |
| *TNFSF10* | 0.7231 | 0.9492 | 0.8740 | 0.8181 | 0.9869 | 0.7529 | **0.0255** | **0.0251** | 0.6756 | 0.9302 |
| *TNFSF14* | 0.6891 | 0.4981 | 0.4479 | 0.5142 | 0.4785 | 0.4460 | 0.3735 | 0.5784 | 0.1050 | 0.0847 |
| *TNFSF18* | 0.7583 | - | 0.3163 | - | 0.3879 | - | 0.6566 | - | 0.5911 | - |
| *TNFSF4* | 0.1304 | 0.2131 | 0.4339 | 0.2833 | 0.1085 | 0.3998 | **0.0197** | 0.2242 | 0.3357 | 0.6791 |
| *TNFSF7* | **0.0301** | **0.0264** | **0.0485** | 0.0676 | **0.0228** | **0.0197** | 0.5170 | 0.2719 | 0.6801 | 0.1901 |
| *TNFSF8* | 0.8824 | 0.9635 | 0.9960 | 0.9858 | 0.4669 | 0.4941 | 0.9044 | 0.9320 | 0.6751 | 0.1057 |
| *TNFSF9* | 0.7851 | 0.7047 | 0.8964 | 0.7350 | 0.5259 | 0.5386 | 0.0857 | 0.0979 | 0.2770 | 0.3745 |
| *TRADD* | 0.4827 | 0.4167 | 0.4761 | 0.2676 | 0.0953 | 0.0904 | 0.9293 | 0.7839 | 0.2638 | 0.1674 |
| *TRAF2* | 0.4805 | - | 0.3609 | - | 0.4829 | - | 0.6174 | - | 0.7727 | - |
| *TRAF5* | 0.3735 | 0.4650 | 0.8792 | 0.8633 | **0.0063** | **0.0218** | 0.3046 | 0.5151 | 0.5825 | 0.9071 |
| *TRAF6* | 0.1280 | 0.3571 | 0.5786 | 0.8129 | 0.2798 | 0.6307 | 0.6952 | 0.6884 | 0.2317 | 0.3416 |
