# Supplementary material for: Common Gene Variants in the Tumor Necrosis Factor (TNF) and TNF Receptor Superfamilies and NF-kB Transcription Factors and Non-Hodgkin Lymphoma Risk
Source: PLoS One. 2009 Apr 24;4(4):e5360. doi: 10.1371/journal.pone.0005360 (PMC2669130; doi:10.1371/journal.pone.0005360)

Supplemental Figure 1. Significance level (p-value) of three-SNP haplotypes within the *TNFSF13B* region, identifying the *TNFSF13B* rs8181791-rs16972216-rs17499386 haplotype (denoted in figure as *TNFSF13B*-07-05-06) as associated with NHL (restricted to non-Hispanic Caucasians). Note: SNP-based analyses demonstrated *TNFSF13B* rs2582869 (denoted as *TNFSF13B*-15) as statistically significant.

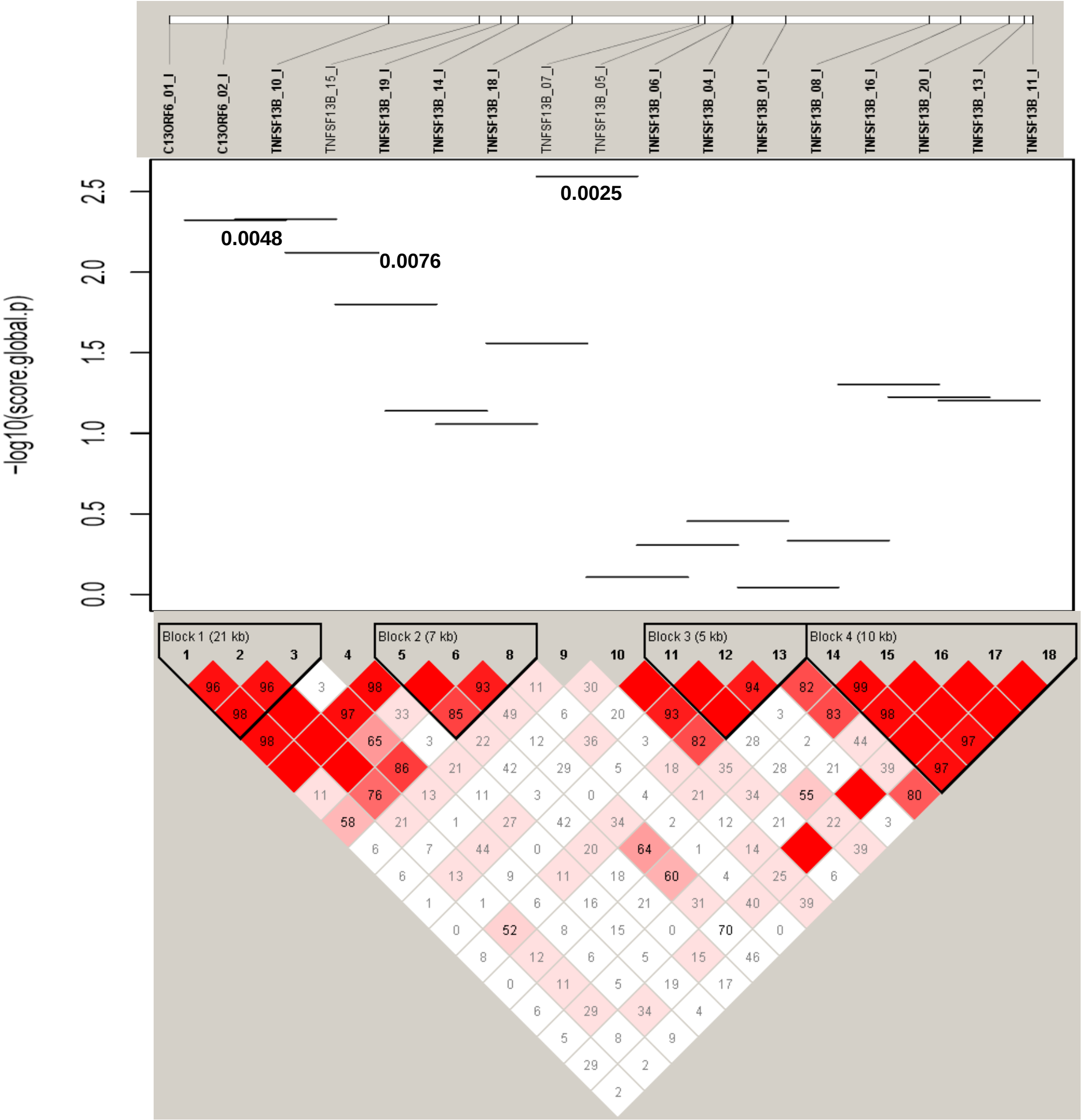

Supplement: Figure S1 — Supplemental Figure 1 (0.06 MB PDF) [file pone.0005360.s007.pdf]
