# Supplementary material for: Common Gene Variants in the Tumor Necrosis Factor (TNF) and TNF Receptor Superfamilies and NF-kB Transcription Factors and Non-Hodgkin Lymphoma Risk
Source: PLoS One. 2009 Apr 24;4(4):e5360. doi: 10.1371/journal.pone.0005360 (PMC2669130; doi:10.1371/journal.pone.0005360)

Supplemental Figure 2. Significance level (p-value) of three-SNP haplotypes within the LTA/TNF region, identifying polymorphisms within *NFKBIL1* significantly associated with NHL where single SNP results were not significant (restricted to non-Hispanic Caucasians). Note: Significant *NFKBIL1* haplotype [rs2857605-rs2239707-rs2230365] denoted in figure as *NFKBIL1*-04-02-01. Previously identified *TNF* G-308A (rs1800629) associated with DLBCL denoted as *TNF*-02. Single SNP association in present analysis demonstrated *LTA* rs2844484 (denoted as LTA-17) as statistically significantly associated with DLBCL.

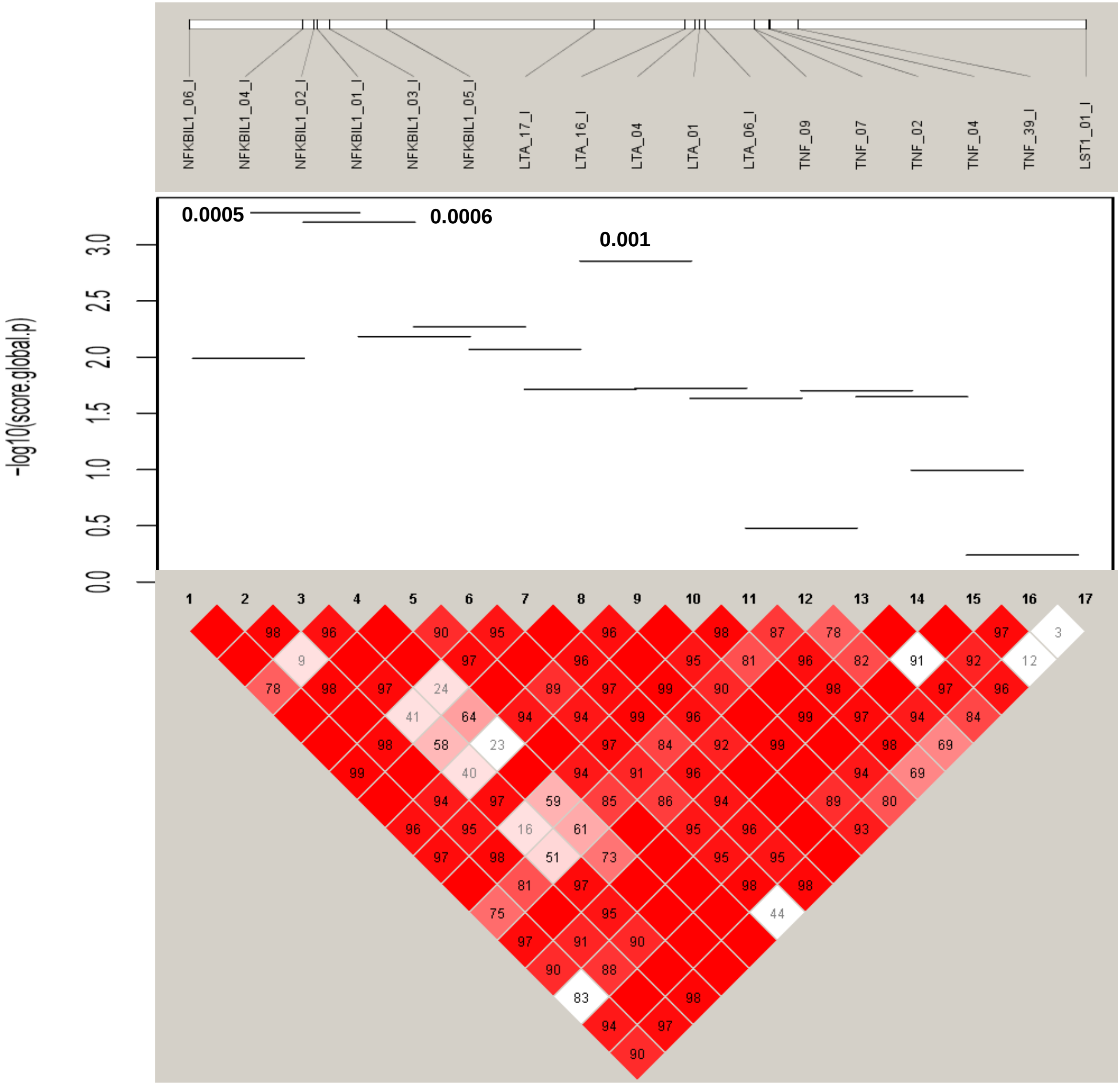

Supplement: Figure S2 — Supplemental Figure 2 (0.06 MB PDF) [file pone.0005360.s008.pdf]
